# Distinct ontogenetic lineages dictate cDC2 heterogeneity

**DOI:** 10.1038/s41590-024-01745-9

**Published:** 2024-02-13

**Authors:** Carlos M. Minutti, Cécile Piot, Mariana Pereira da Costa, Probir Chakravarty, Neil Rogers, Hector Huerga Encabo, Ana Cardoso, Jane Loong, Gilles Bessou, Cyrille Mionnet, Jean Langhorne, Dominique Bonnet, Marc Dalod, Elena Tomasello, Caetano Reis e Sousa

**Affiliations:** 1 Immunobiology Laboratory, The Francis Crick Institute, London, UK; 2 Bioinformatics and Biostatistics, The Francis Crick Institute, London, UK; 3 Haematopoietic Stem Cell Laboratory, The Francis Crick Institute, London, UK; 4 Retroviral Immunology Laboratory, The Francis Crick Institute, London, UK; 5 Aix-Marseille University, Centre National de la Recherche Scientifique, Institut National de la Santé et de la Recherche Médicale, Centre d’Immunologie de Marseille-Luminy, Turing Center for Living Systems, Marseille, France; 6 Malaria Immunology Laboratory, The Francis Crick Institute, London, UK; 7 Present Address: Immunoregulation Laboratory, Champalimaud Research, Champalimaud Centre for the Unknown, Lisbon, Portugal

**Keywords:** Conventional dendritic cells, Myelopoiesis

## Abstract

Conventional dendritic cells (cDCs) include functionally and phenotypically diverse populations, such as cDC1s and cDC2s. The latter population has been variously subdivided into Notch-dependent cDC2s, KLF4-dependent cDC2s, T-bet^+^ cDC2As and T-bet^−^ cDC2Bs, but it is unclear how all these subtypes are interrelated and to what degree they represent cell states or cell subsets. All cDCs are derived from bone marrow progenitors called pre-cDCs, which circulate through the blood to colonize peripheral tissues. Here, we identified distinct mouse pre-cDC2 subsets biased to give rise to cDC2As or cDC2Bs. We showed that a Siglec-H^+^ pre-cDC2A population in the bone marrow preferentially gave rise to Siglec-H^−^ CD8α^+^ pre-cDC2As in tissues, which differentiated into T-bet^+^ cDC2As. In contrast, a Siglec-H^−^ fraction of pre-cDCs in the bone marrow and periphery mostly generated T-bet^−^ cDC2Bs, a lineage marked by the expression of LysM. Our results showed that cDC2A versus cDC2B fate specification starts in the bone marrow and suggest that cDC2 subsets are ontogenetically determined lineages, rather than cell states imposed by the peripheral tissue environment.

## Abstract (web summary)

Reis e Sousa et al. show that cDC2As and cDC2Bs are derived from distinct subsets of bone marrow pre-cDC2s, suggesting that the two lineages are ontogenetically determined.

## Main

Conventional dendritic cells (cDCs) consist of two major subsets, known as cDC1s and cDC2s^1,2^. XCR1^+^ cDC1s are BATF3-dependent^1,2^ and required for inducing cytotoxic T cell responses against many tumor and viral antigens^1^. cDC2s often express CD11b and CD172α (SIRPα), and their differentiation or migratory capacity depends on IRF4 (refs. ^1,2^). Accumulating evidence suggests that cDC2s are required for effective activation of the helper arm of T cell responses^3–12^. However, cDC2s are more heterogenous than cDC1s^3–5,13–15^. Two subgroups of mouse cDC2s were initially defined based on differential requirement for Notch2 or KLF4 for their differentiation^3–5^. Notch2-dependent cDC2s are labeled in *Gpr4* reporter mice and express CD4, CLEC4A4 and endothelial cell-selective adhesion molecule (Esam) in the spleen and CD103 in the intestine^4^. Notch2-independent cDC2s express CLEC12A and are labeled in *Cx3cr1* and *Ccr2* reporter mice and in *Lyz2* fate mapping mice^4^. KLF4-dependent cDC2s are CD172α^+^ and variably express CD24, PD-L2 or MGL-2, depending on the tissue^3^.

More recently, T-bet^+^ and T-bet^−^ cDC2s were found in the spleens of T-bet reporter mice and termed cDC2As and cDC2Bs, respectively^15^. T-bet^+^ cDC2As include Notch2-dependent Esam^+^ cDC2s. The original cDC2B population included a small proportion of cells marked by RORγt fate mapping^15^, later shown to constitute a distinct lymphoid cell type rather than bona fide cDCs^16^. In a further study, KLF4-dependent cDC2s were suggested to correspond to cDC2Bs^17^. Finally, infection or cancer can drive the appearance of cells termed ‘inflammatory cDC2s’ and ‘mature dendritic cells enriched in regulatory molecules’, respectively^12,18,19^. Thus, at present, mouse cDC2s variably include cDC2As, cDC2Bs, Notch-dependent cDC2s, KLF4-dependent cDC2s, inflammatory cDC2s and mature dendritic cells enriched in regulatory molecules. Some of these subpopulations might overlap or correspond to different developmental or activation states of the same DC lineage, while others might represent distinct cDC2 subsets. Adding to the complexity, another population, variably termed transitional DCs (tDCs), AXL^+^ DCs, AS DCs or plasmacytoid-like DCs has been identified in humans and mice^17,20–25^. tDCs are proposed to have a lymphoid origin and recent work suggests that they are part of the plasmacytoid DC lineage, although they can differentiate into cells resembling cDC2As^20,25,26^.

One approach to disentangle this complexity is to study cDC ontogeny. The lifespan of cDCs in tissues is short (3–6 days^27^) such that the cDC tissue network needs to be constantly replenished from bone marrow precursors. The conventional or common DC progenitor (CDP) is the earliest bone marrow cell with DC-restricted potential^1,28^. These CDPs give rise to pre-cDCs, which leave the bone marrow through the blood to seed all tissues and generate terminally differentiated cDC1s and cDC2s^1^. Specification toward the cDC1 or cDC2 lineage starts already at the CDP stage and generates pre-cDC1s and pre-cDC2s^29,30^. The prevailing view is that the latter then diversify by acquiring distinct phenotypic or functional traits in different tissue niches or under different inflammatory conditions^15,31^. In line with this notion, retinoic acid supports the differentiation of Notch2-dependent cDC2s in the intestine and spleen^32,33^; type 3 innate lymphoid cells (ILC3s) in the spleen promote the differentiation of cDC2As through the production of lymphotoxin^34^. However, it is possible that cDC2 diversity specification might occur at the pre-cDC level in the bone marrow and that signals in tissue are permissive rather than instructive.

In this study, we used a binary definition of cDC2s, splitting them, as proposed^15^, into T-bet^+^ cDC2As and T-bet^−^ cDC2Bs. We showed that cDC2As and cDC2Bs in mice at steady state phenotypically encompass the previously described Notch-dependent and KLF4-dependent cDC subsets. Notably, we found that pre-cDC2s in the bone marrow could already be divided into two subtypes that preferentially gave rise to cDC2As or cDC2Bs. The identification of biased pre-cDC2A and pre-cDC2B populations in mouse and human bone marrow supports the notion that cDC2As and cDC2Bs represent distinct ontogenetic lineages.

## Results

### Notch2-dependent and KLF4-dependent cDC2s correspond to cDC2As and cDC2Bs

**Extended Data Fig. 1:**
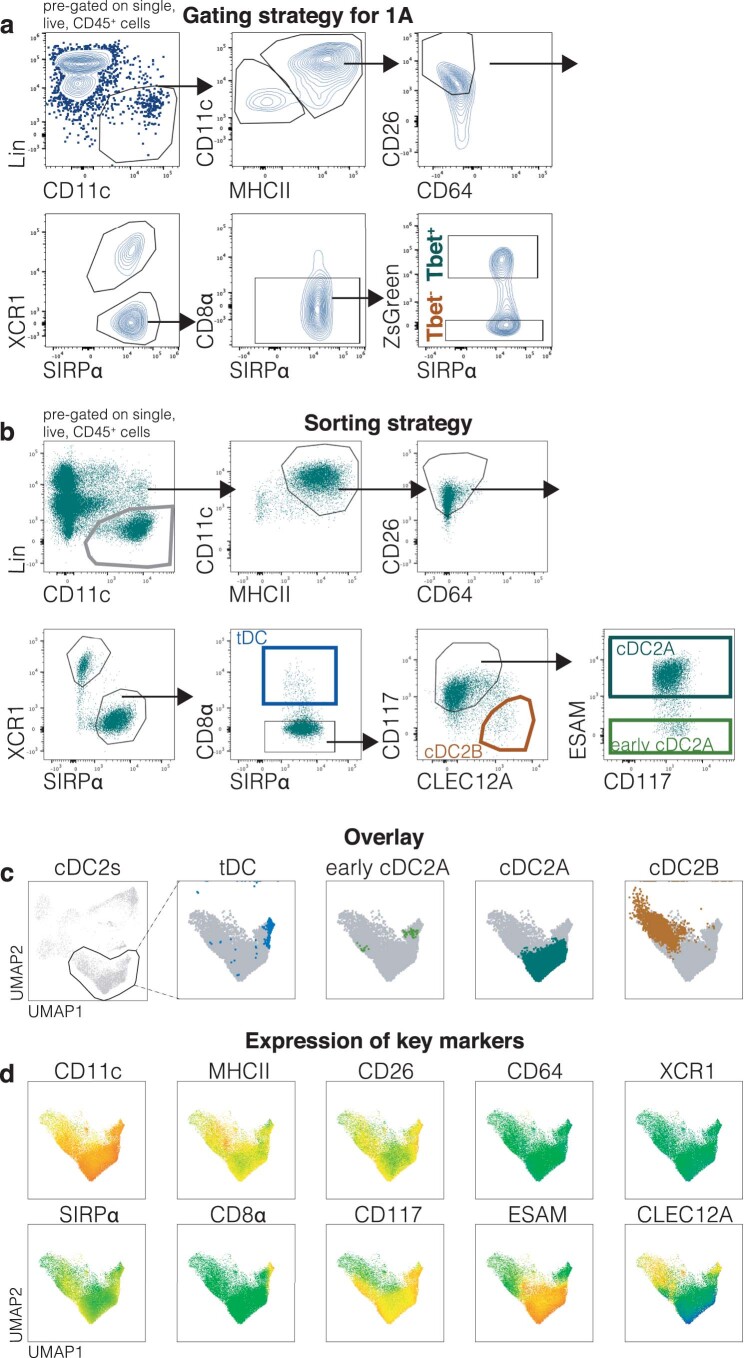
Spleen cDC2 gating strategy. **a**, Gating strategy used in 1a. Leftmost panel shows cells pre-gated on single, live, CD45^+^. The lineage cocktail includes antibodies against CD3, Ly6G, SiglecF, B220, CD19, NK1.1, Ly6D, and Ter119. Lin^−^ CD11c^+^ and MHC-II^+^ cells are initially selected, after which CD26^hi^ and CD64^−/int^ cDCs are divided into cDC1s (XCR1^+^) and cDC2s (SIRPα^+^, CD64^−/int^). After excluding CD8α^+^ tDCs, cDC2s are split into ZsGreen^+^ and ZsGreen^-^ for further analysis. Arrows denote gate hierarchy. **b**, Sorting strategy for spleen cDC2s. Leftmost panel shows cells pre-gated on single, live, CD45^+^. The lineage cocktail includes antibodies against CD3, Ly6G, SiglecF, B220, CD19, NK1.1, Ly6D, and Ter119. Lin^-^ CD11c^+^ and MHC-II^+^ cells are initially selected, after which CD26^hi^ and CD64^−/int^ cDCs are divided into cDC1s (XCR1^+^) and cDC2s (SIRPα^+^, CD64^−/int^). After excluding CD8α^+^ tDCs (dark blue), cDC2As (teal) and cDC2Bs (orange) are identified using ESAM and CLEC12A, respectively. Arrows denote gate hierarchy. **c**, Manual gates from B are overlaid onto a UMAP (same as 1b) of the Lin^-^ CD11c^+^ cells (from the first gate of the manual strategy in a). The UMAP was generated on the basis of CD11c, MHC-II, CD26, CD64, CD88, XCR1, SIRPα, ESAM, CLEC12A, CD11b, CD43, CD135, CD117, Ly6C, and CD8α. cDC2s in the leftmost UMAP are gated and zoomed in the following panels, where cDC2 subsets gated manually are overlaid. See also Fig. 1b. **d**, The expression of key markers used to define different cDC and tDC subpopulations in the UMAPs is shown in the form of heatmaps. Expression levels are represented as a colour gradient from low (blue) to high (orange).

We phenotyped cDCs from mice in which T-bet expression is reported by ZsGreen (hereafter T-bet-ZsGreen mice)^35^. We defined cDCs as Lin (CD3, Ly6G, Siglec-F, B220, CD19, Ly6D, NK1.1 and Ter119)^−^ CD64^−/lo^CD11c^+^ major histocompatibility complex (MHC) class II (MHC-II)^+^CD26^+^, and cDC1 and cDC2 as XCR1^+^ and SIRPα^+^, respectively^12,36^. tDCs within the cDC2 gate were identified as CD8α^+^ cells^20,25,26^ (Extended Data Fig. 1a,b). To mark previously identified cDC2 populations, we used Esam for Notch-dependent cDC2s^4^, CD24 and MGL-2, programmed cell death 1 ligand 2 (PD-L2) for KLF4-dependent cDC2s^3^, T-bet-ZsGreen for cDC2As^15^ and CLEC12A for cDC2Bs^15^.

**Fig. 1:**
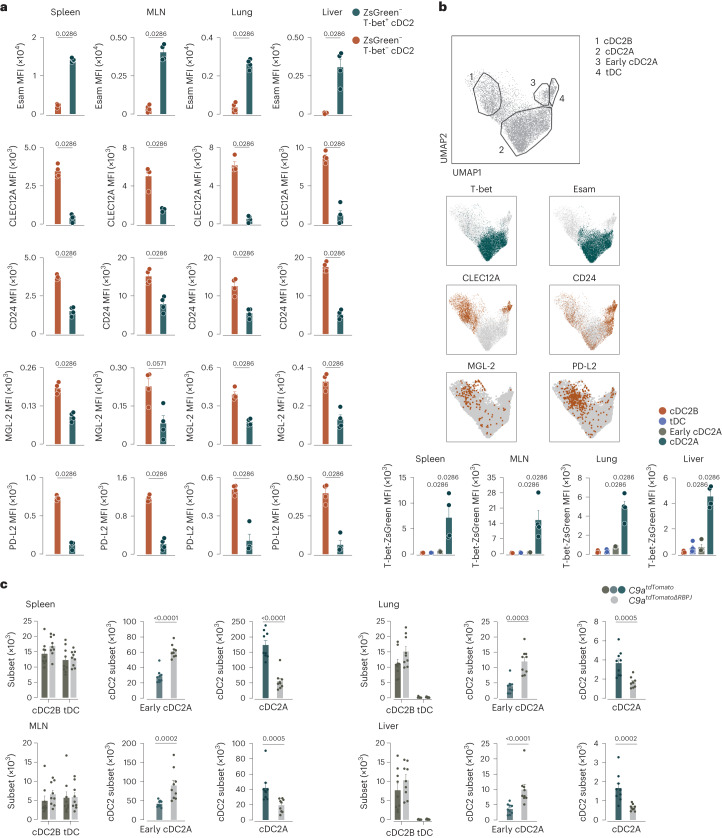
cDC2As include Notch2-dependent cDC2s whereas KLF4-dependent cDC2s correspond to cDC2Bs. **a**, Flow cytometry analysis showing expression of Esam, CLEC12A, CD24, MGL-2 and PD-L2 on T-bet-ZsGreen^+^ (cDC2A) and T-bet-ZsGreen^−^ (cDC2B) cDC2s from the spleen, MLN, lung and liver in T-bet-ZsGreen mice. **b**, Representative UMAP of flow cytometry data of spleen cells from Tbx21-ZsGreen mice that fall into the cDC2 gate generated from Lin^−^CD11c^+^ cells using the expression of CD11c, MHC-II, CD26, CD64, CD88, XCR1, SIRPα, Esam, CLEC12A, CD11b, CD43, CD135, CD117, Ly6C and CD8α indicating the cDC2Bs, cDC2As, early cDC2As and tDCs (top) overlays of T-bet-ZsGreen^+^, Esam^+^, CLEC12A^+^, CD24^+^, MGL-2^+^ or PD-L2^+^ cDC2s onto the UMAP (middle) and ZsGreen mean fluorescence intensity (MFI—after subtracting the autofluorescence background) in cDC2Bs, cDC2As, early cDC2As and tDCs identified in the UMAP (bottom). **c**, Flow cytometry analysis showing the quantification of spleen, MLN, lung and liver cDC2Bs, cDC2As, early cDC2As and tDCs from *C9a*^*tdTomato*^ and *C9a*^*tdTomatoΔRBPJ*^ mice. Each dot represents one mouse (*n* = 4 in **a**,**b** and *n* = 9 in **c**). Data are from one of two (**a**,**b**) or a pool of two (**c**) experiments (mean ± s.e.m.). A two-tailed Mann–Whitney *U*-test was used to compare groups (in **b**, the comparison is relative to cDC2B). *P* values are indicated above the graphs.

**Extended Data Fig. 2:**
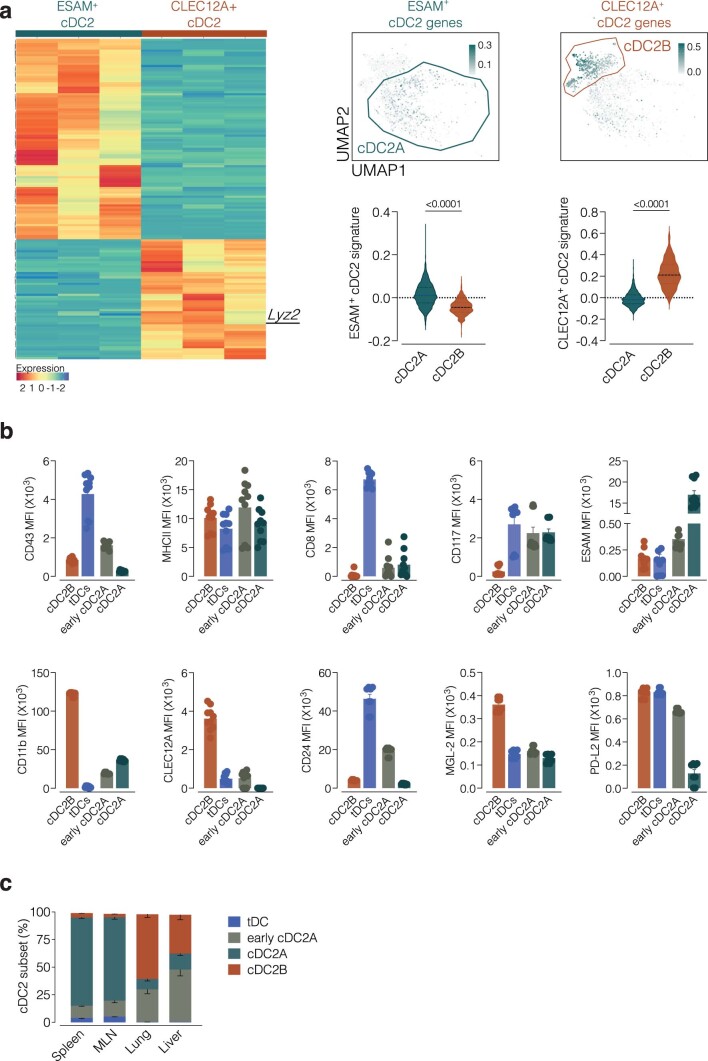
Validation of spleen cDC2 gating strategy. **a**, (Left) Heatmap representation of the top differentially expressed genes (an adjusted p value of < 0.05) from a new bulk RNAseq analysis of the two cDC2 populations (ESAM^+^ cDC2s and CLEC12A^+^ cDC2s) sorted using the gating strategy shown in Extended Data Fig. 1b (PCA is shown later in Extended Data Fig. 5a). Expression levels are represented as a colour gradient from low (blue) to high (orange). Each column represents a sample coming from a pool of 5 mice. Note that the expression of *Esam*, *Clec12a* and *Tbx21* was either not detected or not significant in the statistical analysis. (Right) Feature plots representing the score of DEGs from a (used as signatures) of ESAM^+^ cDC2s and CLEC12A^+^ cDC2s overlaid onto a UMAP of cDC2As and cDC2Bs generated from the Brown et al scRNAseq dataset^15^. Expression levels are shown as a gradient from low (light grey) to high (teal). The quantification of the scores is shown on the bottom in the form of violin plots. **b**, (from left to right and top to bottom) FACS analysis showing CD43, MHC-II, CD8α, CD117, ESAM, CD11b, CLEC12A, CD24, MGL-2 and PD-L2 expression on spleen cDC2 and tDC populations (identified from UMAP gates as shown in 1b and Extended Data Fig. 1c, d). **c**, FACS analysis showing the percentage of different populations (identified as shown in 1b and Extended Data Fig. 1c, d) in the indicated tissues. Data in (c-d) are a pool of two experiments (n = 8) (means ± SEM, median ± IQR for violin plot). Each dot in b represents one mouse (n = 8). Mann-Whitney test (two-tailed) was used to compare cDC2As and cDC2Bs in A. P values are indicated on top of the graphs.

We started by splitting cDC2s into ZsGreen^+^ and ZsGreen^−^ (Extended Data Fig. 1a). This revealed a marked overlap between the expression of Esam and T-bet-ZsGreen in all the tissues analyzed (spleen, mesenteric lymph node (MLN), lung and liver; Fig. 1a). In contrast, T-bet-ZsGreen^−^ cDC2Bs showed preferential expression of CLEC12A and variable expression of CD24, MGL-2 and PD-L2 (Fig. 1a). Thus, using marker analysis, T-bet-ZsGreen^+^ cDC2As included Notch2-dependent cDC2s whereas T-bet-ZsGreen^−^ cDC2Bs corresponded to KLF4-dependent cDC2s^15,17^. Uniform manifold approximation and projection (UMAP) dimension reduction analysis using all markers except T-bet-ZsGreen to drive cluster segregation, together with bulk RNA sequencing (RNA-seq), indicated that Esam and CLEC12A accurately defined cDC2As and cDC2Bs, respectively, independently of T-bet-ZsGreen labeling (Fig. 1b, Extended Data Figs. 1b–d and 2a, and Supplementary Table 1). We found a relatively small cluster of tDCs (cluster 4) that was CD8α^+^ CD11b^−^ (Fig. 1b and Extended Data Fig. 2b) and a CD8α^−^ cluster that segregated from tDCs (cluster 3) (Fig. 1b and Extended Data Fig. 2b).

To refine cDC2A and cDC2B identification, we used *Clec9a*^*Cre*^*Rosa26*^*LSL-tdTomato*^*Rbpj*^*loxP*^^/*loxP*^mice (*C9a*^*tdTomatoΔRBPJ*^) that lack Notch signaling in the cDC lineage and compared them to *Clec9a*^*Cre*^*Rosa26*^*LSL-tdTomato*^ controls (*C9a*^*tdTomato*^). The number of cDC2As, but not cDC2Bs (as defined by the UMAP clusters), was reduced in *C9a*^*tdTomatoΔRBPJ*^ mice in all organs analyzed (Fig. 1c). *C9a*^*tdTomatoΔRBPJ*^ mice also displayed an increase in cluster 3 (CD8α^−^CD117^+^Esam^−^) across all tissues (Fig. 1c and Extended Data Fig. 2b), suggesting that these cells were immediate precursors of cDC2As whose terminal differentiation was arrested in the absence of Notch signals (hereafter early cDC2As)^4,5^. CD8α^+^ tDCs were only found in spleen and MLN but were not decreased in *C9a*^*tdTomatoΔRBPJ*^ mice (Fig. 1c and Extended Data Fig. 2c). Together with reports showing that cDC2Bs, but not cDC2As, are KLF4-dependent^17^, our data suggested that the overall heterogeneity of cDC2s can be distilled down to two main Notch-dependent T-bet^+^ cDC2A and Notch-independent T-bet^−^ cDC2B branches and states of differentiation along them.

### Single-cell RNA-seq defines cDC2 heterogeneity at the pre-cDC2 level

**Fig. 2:**
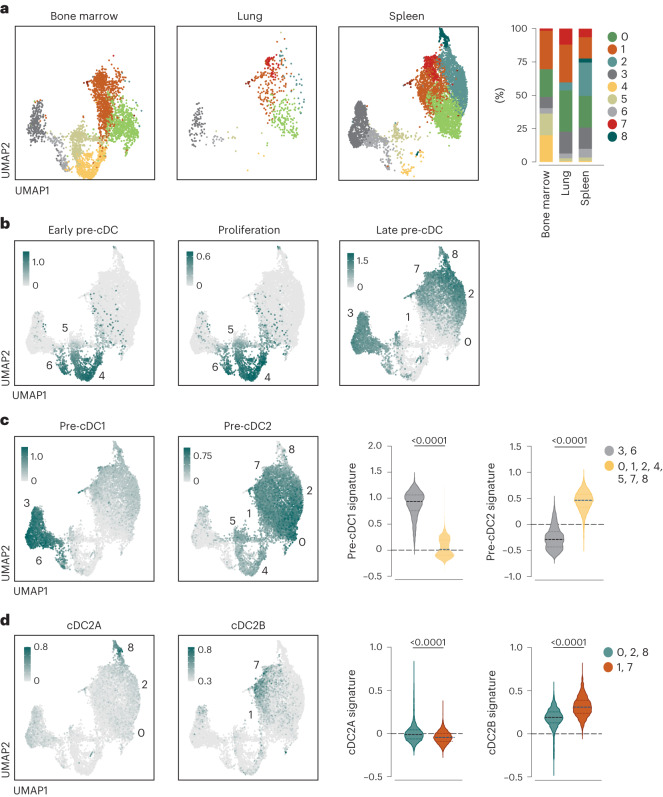
cDC heterogeneity can be recapitulated at the pre-cDC level. **a**, UMAPs displaying scRNA-seq analysis of pre-cDCs sorted as shown in Extended Data Fig. 3e from the bone marrow (2,649 cells), spleen (4,371 cells) and lung (358 cells) with unsupervised clustering (each sample is a pool of six mice). The proportion of the nine clusters identified in the UMAPs for each organ is shown on the right. **b**, Representative plots depicting the score for the gene signatures (refs. ^15,30^ and Supplementary Table 9) of proliferation (middle) and early (left) or late (right) pre-cDC projected onto the concatenated UMAP space. Expression levels are shown as a gradient from low (light gray) to high (teal). **c**, Feature plots depicting the score for the gene signatures (refs. ^15,30^ and Supplementary Table 9) of pre-cDC1s and pre-cDC2s on the concatenated UMAP, and violin plots for the scores within the 3 and 6, and 0, 1, 2, 4, 5, 7 and 8, cluster groups. **d**, Feature plots depicting the score for the gene signatures (refs. ^15,30^ and Supplementary Table 9) of cDC2As and cDC2Bs on the concatenated UMAP, and violin plots for the scores within the 0, 2 and 8, and 1 and 7, cluster groups. Expression levels are shown as a gradient from low (light gray) to high (teal). In **c**,**d**, a two-tailed Mann–Whitney *U*-test was used for comparison (median ± the interquartile range (IQR)). *P* values are indicated above the graphs.

**Extended Data Fig. 3:**
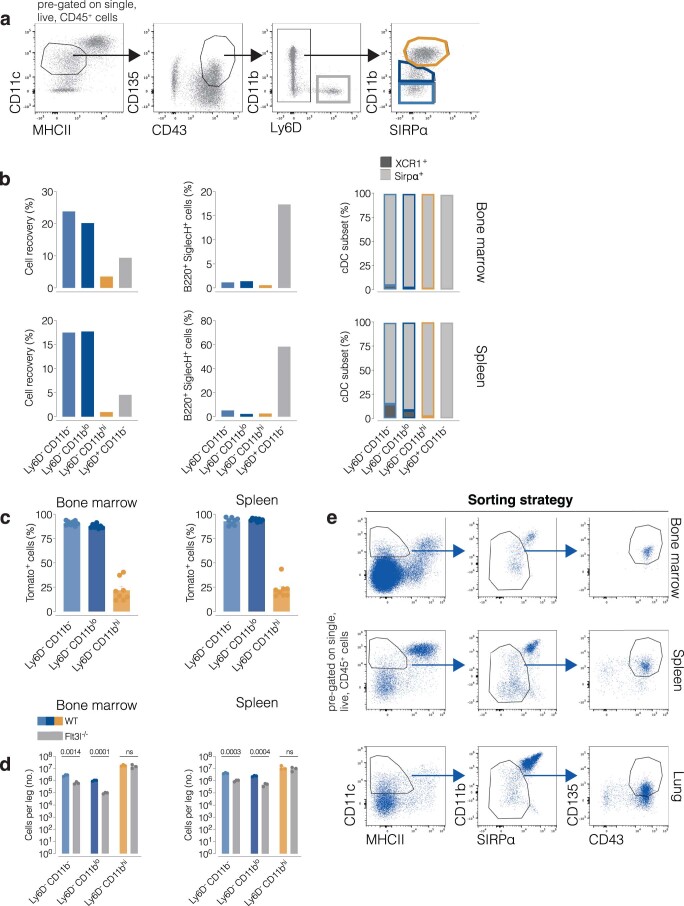
Validation of gating strategy for sorting total pre-cDC populations from tissues. **a**, Sorting strategy for pre-cDCs (and other precursor cells to ascertain which ones are bona fide pre-cDCs). Live single cells from spleen or bone marrow cell suspensions negative for lineage markers (CD3, Ly6G, SiglecF, B220, CD19, NK1.1, and Ter119) and positive for CD45.2 were analysed as follows: CD11c^+^ MHC-II^−/lo^ were selected, from this gate, the CD135^+^ CD43^+^ cells contained the pre-cDCs and other contaminants. CD135^+^ CD43^+^ cells contained two populations: Ly6D^+^ and Ly6D^−^ cells. The Ly6D^+^ cells were directly sorted as one population (grey gate). The Ly6D^-^ cells were further split into three subpopulations that were sorted as shown on the fourth panel: CD11b^-^ (light blue gate), CD11b^lo^ (dark blue gate) and CD11b^hi^ (orange gate). Arrows denote gate hierarchy. **b**, The populations highlighted in panels 3 and 4 were sorted from the bone marrow (top) or spleen (bottom) and cultured for 3 days with OP9-DL1 stromal cells in the presence of Flt3L. Data are FACS analysis showing the % recovery after differentiation and frequency of cDC subsets and plasmacytoid cells among the progeny. These populations were analysed using manual gating and were defined as: single, live, CD45.2^+^, CD11c^+^ MHC-II^+^ cells. cDC1s are defined as XCR1^+^ while cDC2s express SIRPα. The right panel shows the cDC1/cDC2 subset distribution of progeny from the sorted cells after differentiation. **c**, FACS analysis showing TdTomato labelling of the indicated cell populations from the bone marrow or spleen of *C9a*^*TdTOM*^ mice gated as shown in a. **d**, FACS analysis showing the abundance of the indicated cell populations (gated as shown in a) in the bone marrow and spleens of WT and Flt3L-deficient mice. **e**, Refined gating strategy used to sort total pre-cDCs from tissues taking into account the results from a-d. In this sorting strategy, pre-cDCs are identified as leukocytes that are negative for many lineage-restricted markers (CD3, Ly6G, SiglecF, B220, CD19, Ly6D, NK1.1, and Ter119), as well as negative/low for surface expression of MHC-II, CD11b and SIRPα, but positive for CD11c, CD135, and CD43. Each dot represents one mouse (n = 3 in b and d and 8 in c). Data are from one out of two experiments (b, d) or a pool of two (c) (means ± SEM). Mann-Whitney test (two-tailed) was used to compare WT and *Flt3l*^*−/−*^ mice in (d). P values are indicated on top of the graphs.

We next identified pre-cDCs in tissues using a protocol developed for isolating lung pre-cDCs^18^. We gated on Lin^−^CD11c^+^MHC-II^−/lo^CD11b^−/lo^SIRPα^−^CD135^+^CD43^+^ cells while excluding Ly6D^+^ cells (precursors of both plasmacytoid cells^37,38^ and tDCs^25^) and CD11b^hi^SIRPα^+^CD16/32^+^ cells (monocyte-like cells and DC3 progenitors^39^) (Extended Data Fig. 3a). Using in vitro differentiation assays (Extended Data Fig. 3b), fate mapping (Extended Data Fig. 3c) and in vivo Fms-like tyrosine kinase 3 ligand (Flt3L) dependence (Extended Data Fig. 3d), we confirmed that the gating strategy identified bona fide pre-cDCs in the bone marrow and spleen, as previously shown for the lung^18^. We used the gating strategy (Extended Data Fig. 3e) to sort pre-cDCs from the bone marrow, spleen and lung of C57BL/6J wild-type (WT) mice. We performed single-cell RNA-seq (scRNA-seq) analysis on 2,649 bone marrow, 4,371 spleen and 358 lung-sorted pre-cDCs after excluding a small number of dying cells and contaminants (identified using immune cell transcriptome profiles; https://www.immgen.org/) (Fig. 2a). We integrated the three tissues (bone marrow, spleen and lung) and generated a UMAP that identified nine clusters that, although varying in proportion, overlapped across all tissues (Fig. 2a). Therefore, we concatenated the cells from all tissues and used published gene signatures^15,30^ to annotate the UMAP clusters. This approach indicated that clusters 4, 5 and 6 corresponded to proliferative early pre-cDCs (Fig. 2b). They were enriched in bone marrow (Fig. 2a,b), which is consistent with the fact that they originate in that tissue. Clusters 0 and 1 probably represented more differentiated pre-cDCs about to leave the bone marrow^40^ or pre-cDCs that recently colonized peripheral tissues (Fig. 2b). Clusters 3, 2, 7 and 8 (late pre-cDCs) were overrepresented in peripheral tissues (Fig. 2a,b), where pre-cDCs complete differentiation into cDCs^1^. Overall, pre-cDCs segregated into two groups: one consisting of clusters 3 and 6 with a gene expression signature of pre-cDC1s/cDC1s; and one consisting of clusters 0, 1, 2, 4, 5, 7 and 8 and similar in gene expression to pre-cDC2s/cDC2s (Fig. 2c)^29,30^. We did not identify any cluster that appeared uncommitted at the level of the gene expression signature (Fig. 2c), as expected^29,30^. Pre-cDC2s were relatively more heterogenous than pre-cDC1s (seven compared to two clusters) (Fig. 2c). Within the late pre-cDC2s clusters, there were two broad groups: clusters 0, 2 and 8 showed increased similarity in gene expression profile to cDC2A; clusters 1 and 7 expressed more genes in common with cDC2B (Fig. 2d). These data suggested that subdivision of cDC2s into cDC2As and cDC2Bs could be recapitulated at the level of their pre-cDC precursors using gene expression profiling.

### Pre-cDC2s are biased toward the cDC2A or cDC2B fate

**Fig. 3:**
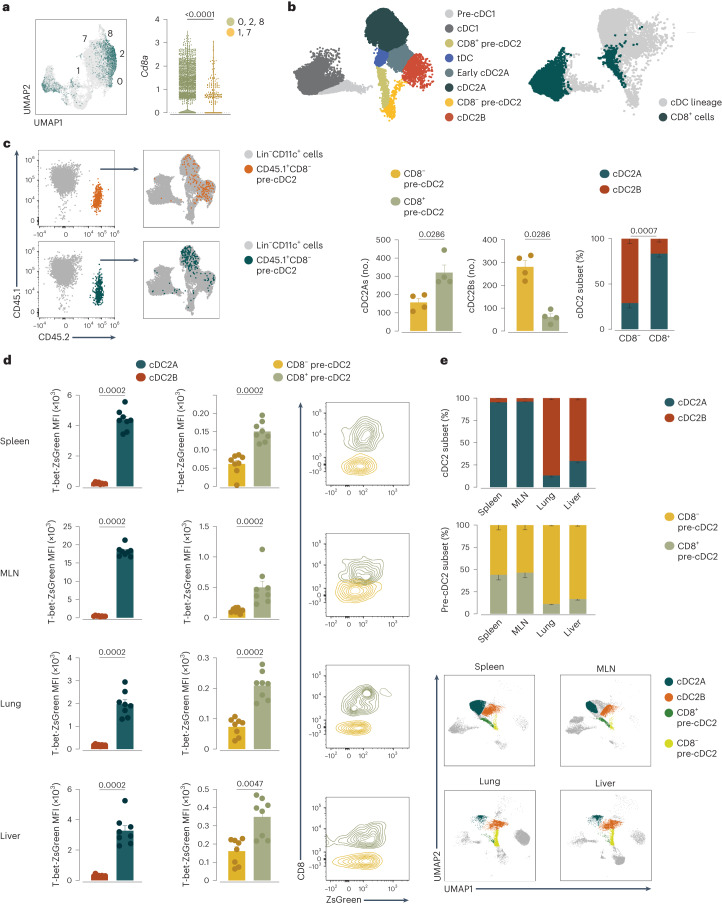
Peripheral pre-cDC2s are biased toward the cDC2A or cDC2B fate. **a**, Feature plot (left) and violin plot (right) showing *Cd8a* expression on the concatenated UMAP or in cluster groups 0, 2 and 8, or 1 and 7, as in Fig. 2b–d. **b**, Representative UMAP of flow cytometry analysis of splenic pre-cDC and cDC populations generated on CD11c^+^Lin^−^ cells using CD11c, MHC-II, CD26, CD64, CD88, XCR1, SIRPα, Esam, CLEC12A, CD11b, CD43, CD135, CD117, Ly6C and CD8α (left), and CD8α^+^ cells overlaid onto the UMAP (right). **c**, CD45.2^+^ cDC2s (derived from CD8α^−^ or CD8α^+^ pre-cDC2s) recovered from the spleen of CD45.1 recipient mice overlaid onto a UMAP representing the cDC lineage of the host (left) and flow cytometry analysis showing the number and percentage of WT CD45.2 Esam^+^ cDC2As and CLEC12A^+^ cDC2Bs recovered from the spleen of WT CD45.1 recipient mice 3 days after transfer of the CD8α^−^ and CD8α^+^ CD45.2 pre-cDC2s populations (right). Populations are annotated in **b**. **d**, ZsGreen MFI (after subtracting the autofluorescence background) in cDC2As and cDC2Bs or CD8α^−^ or CD8α^+^ pre-cDC2s from T-bet-ZsGreen mice and representative flow cytometry plots with overlaid CD8α^+^ pre-cDC2s and CD8α^−^ pre-cDC2s in the spleen, MLN, lung and liver depicting T-bet-ZsGreen expression (fluorescence intensity) in each pre-cDC2 population. **e**, Percentage of cDC2As and cDC2Bs or CD8α^−^ or CD8α^+^ pre-cDC2s in the spleen, MLN, lung and liver and representative UMAP for the spleen, MLN, lung and liver showing the clusters containing cDC2As and cDC2Bs or CD8α^−^ or CD8α^+^ pre-cDC2s. In **c**,**d**, each dot represents one mouse (*n* = 4 in **c** and *n* = 8 in **d**,**e**); data were pooled from two experiments (mean ± s.e.m.; median ± IQR for the violin plot). In **c**–**e**, quantifications come from the UMAPs (as shown in **b** and Extended Data Fig. 4c–e). A two-tailed Mann–Whitney *U*-test was used for comparison. *P* values are indicated above the graphs.

**Extended Data Fig. 4:**
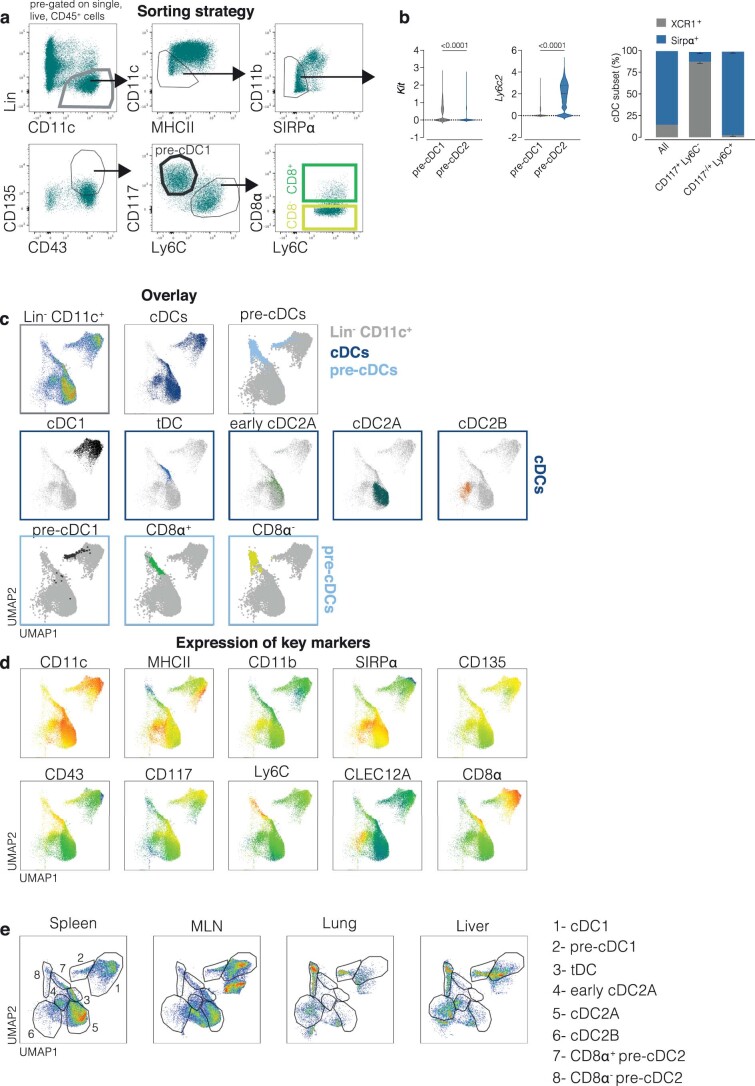
Pre-cDC subset identification in the spleen, MLN, lung and liver. **a**, Sorting strategy for spleen pre-cDC subsets. Leftmost panel has been pre-gated on single, live, CD45^+^, and lineage^−^ spleen cells. The lineage cocktail includes antibodies against CD3, Ly6G, SiglecF, B220, CD19, NK1.1, Ly6D, and Ter119. CD117 and Ly6C are used to identify pre-cDC1s (dark grey) and pre-cDC2s, respectively. CD8α labels the putative pre-cDC2As (light green) whereas the putative pre-cDC2Bs are CD8α^−^ (yellow). Arrows denote gate hierarchy. **b**, (left) Violin plots showing the expression of *Kit* and *Ly6c2* in pre-cDC1s (clusters 3 and 6) or pre-cDC2s (clusters 0, 1, 2, 4, 5, 7, 8) from scRNAseq analysis (UMAP of data concatenated from all tissues). (right) Total pre-cDCs or the indicated subsets were sorted from spleen (sorting strategy as in Extended Data Fig. 4a) and cultured for 3 days with OP9-DL1 stromal cells in the presence of Flt3L. The progeny after differentiation was analysed by FACS for cDC subset distribution. Cells were analysed using manual gating and defined as: single, live, CD45.2^+^, CD11c^+^ MHC-II^+^. cDC1s are defined as XCR1^+^, whereas cDC2s express SIRPα. **c**, Manual gates as in Extended Data Fig. 4a for pre-cDCs and as in Extended Data Fig. 1b for cDC were overlaid onto a UMAP analysis of the spleen (same as 3b). Colours for pre-cDCs correspond to the gates in a. The UMAP was generated using the Lin^-^ CD11c^+^ cells from the first gate of the manual strategy in a, and using the following markers: CD11c, MHC-II, CD26, CD64, CD88, XCR1, SIRPα, ESAM, CLEC12A, CD11b, CD43, CD135, CD117, Ly6C, and CD8α. **d**, The expression of key markers used to define different pre-cDC subpopulations in the UMAPs (from spleen in 3b) is shown in the form of heatmaps. Expression levels are represented as a colour gradient from low (blue) to high (orange). **e**, Representative UMAP analysis from the spleen, MLN, lung and liver. UMAP was generated as in b. Ungated cells are migratory cDC1s and cDC2s, and probably DC3s and were not analysed in this study. In b (right) data are a pool of two experiments (n = 4) (means ± SEM and median ± IQR for violin plot). Mann-Whitney test (two-tailed) was used for comparisons. P values are indicated on top of the graphs.

**Extended Data Fig. 5:**
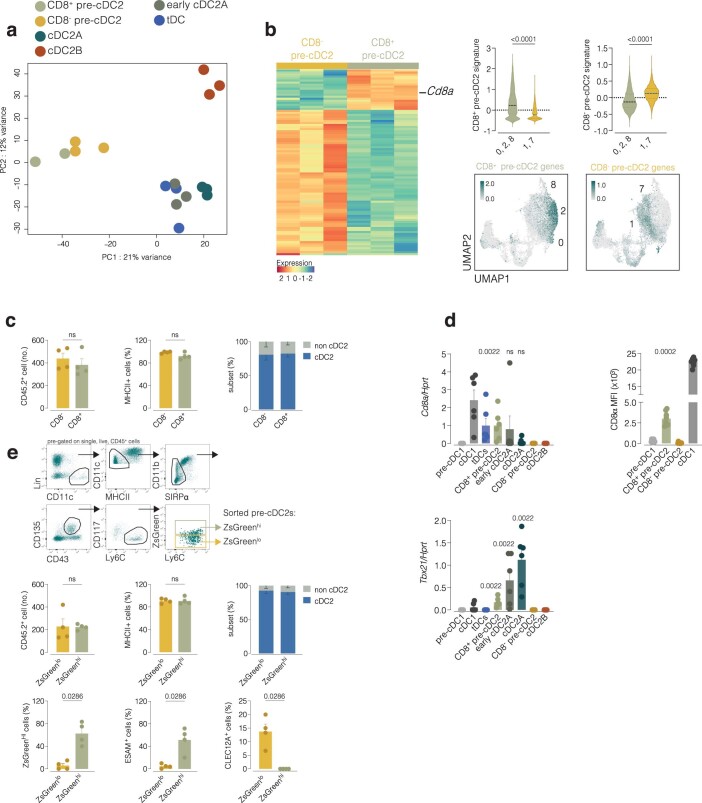
Validation of the strategy to identify splenic pre-cDC2 subsets. **a**, PCA of all expressed genes from a bulk RNAseq (same as Extended Data Fig. 2a) of the indicated populations sorted from spleen as shown in Extended Data Figs. 1b and 4a. **b**, (left) Heatmap representation of the top DEGs (an adjusted p value of <0.05) defining CD8α^−^ pre-cDC2 and CD8α^+^ pre-cDC2 analysed by bulk RNAseq (same analysis as a). Expression levels are represented as a colour gradient from low (blue) to high (orange). Each column represents a sample coming from a pool of 5 mice. (right) Feature plots representing the score of the CD8α^−^ and CD8α^+^ pre-cDC2 signatures (signatures are the list of DEGs from the heatmap on the left) projected on the concatenated UMAP. Expression levels are shown as a gradient from low (light grey) to high (teal). The quantification of the scores is shown on top of the plots. **c**, FACS analysis showing (left) recovery (number of cells), (middle) differentiation (upregulation of MHC-II) and (right) cDC2 specification (upregulation of SIRPα) of WT CD45.2 cells recovered from spleens of WT CD45.1 recipient mice 3 days after transfer of the indicated CD45.2 pre-cDC2s populations (1–4x10^4^ cells sorted as shown in Extended Data Fig. 4a). **d**, qRT-PCR analysis showing expression of *Cd8a* (top left) and *Tbx21* (bottom) in the indicated spleen cell populations (FACS-sorted as shown in Extended Data Figs. 1b and 4a). (top right) Flow cytometric quantification of CD8α expression in the indicated populations (gated as in Extended Data Fig. 4c–e). **e**, FACS analysis of CD45.2 cells recovered from spleens of CD45.1 mice 3 days after receiving the indicated CD45.2 pre-cDC2s populations from T-bet- ZsGreen mice (1–4 × 10^4^ cells sorted as shown on top – negative gate was set using a WT counterpart). Data are: (top left) recovery (number of cells), (top middle) differentiation (upregulation of MHC-II), (top right) cDC2 specification (upregulation of SIRPα), (bottom left) % ZsGreen^+^, (bottom middle) % ESAM^+^ and (bottom right) % CLEC12A^+^ cells. Each dot represents one mouse, and data are a pool of two experiments (n = 4 in c and e and 6 in d) (means ± SEM, median ± IQR for violin plot). Mann-Whitney test (two-tailed) was used for comparisons. P values are indicated on top of the graphs. In d CD8α^+^ pre-cDC2 were compared against CD8α^−^ pre-cDC2, and cDC2A (and early cDC2A) against cDC2B.

We used Comet, a tool for predicting cell population surface markers from scRNA-seq data^41^, to design a strategy to identify putative pre-cDC subsets using flow cytometry. Comet identified markers previously used to distinguish pre-cDC1s (CD117 and CD24) from pre-cDC2s (Ly6C and CD115, among others)^29,30^ (Supplementary Table 2), the accuracy of which we confirmed using in vitro differentiation assays (Extended Data Fig. 4a,b). Comet further identified CD8α as a marker for the putative pre-cDC2As, in addition to marking cDC1s and tDCs in some tissues (Fig. 3a, Extended Data Fig. 4c–e and Supplementary Table 2). Using flow cytometry, we confirmed that Ly6C^+^ pre-cDC2s encompassed CD8α^−^ and CD8α^+^ cells (Fig. 3b and Extended Data Fig. 4a,c–e). UMAP analysis of Lin^−^ spleen cells stained for multiple cDC and pre-cDC markers positioned CD8α^−^ pre-cDC2s on a branch leading to cDC2B, and CD8α^+^ pre-cDC2s on a distinct one leading to cDC2A (Fig. 3b and Extended Data Fig. 4c,d). We sorted spleen CD8α^+^ pre-cDC2s and CD8α^−^ pre-cDC2s (Extended Data Fig. 4a) and performed bulk RNA-seq analysis (Extended Data Fig. 5a). Differentially expressed genes (DEGs) from either population (Supplementary Table 3) were used as a gene signature, which when overlaid on the earlier scRNA-seq UMAP analysis (Extended Data Fig. 5b), indicated that CD8α was indeed able to segregate putative precursors of cDC2As (CD8α^+^ pre-cDC2s) and cDC2Bs (CD8α^−^ pre-cDC2s) in mouse spleen (Extended Data Fig. 5b). This analysis also indicated that although tDCs express CD8α, their gene expression profile was distinct from that of CD8α^+^ pre-cDC2s (Extended Data Fig. 5a).

To directly test precursor–product relationships, we isolated splenic CD8α^−^ and CD8α^+^ pre-cDC2s from CD45.2 mice and transferred them into sublethally irradiated CD45.1 recipients. We excluded Ly6D^+^ cells to exclude precursors of plasmacytoid cells or tDCs, and CD11b^hi^SIRPα^+^ cells to exclude monocyte-like cells and DC3 progenitors. Analysis of splenic cDCs 3 days after transfer showed that both CD45.2^+^CD8α^−^ pre-cDC2s and CD45.2^+^CD8α^+^ pre-cDC2s had differentiated into SIRPα^+^ cDC2s to a comparable extent (Extended Data Fig. 5c). However, the CD8α^−^ pre-cDC2s preferentially generated CLEC12A^+^ cDC2Bs whereas the CD8α^+^ pre-cDC2s predominantly became Esam^+^ cDC2As (Fig. 3c). Thus, CD8α, a marker associated with cDC1s and tDCs, was also expressed by splenic pre-cDC2As and could be used to differentiate them from splenic pre-cDC2Bs (Extended Data Fig. 5d).

**Extended Data Fig. 6:**
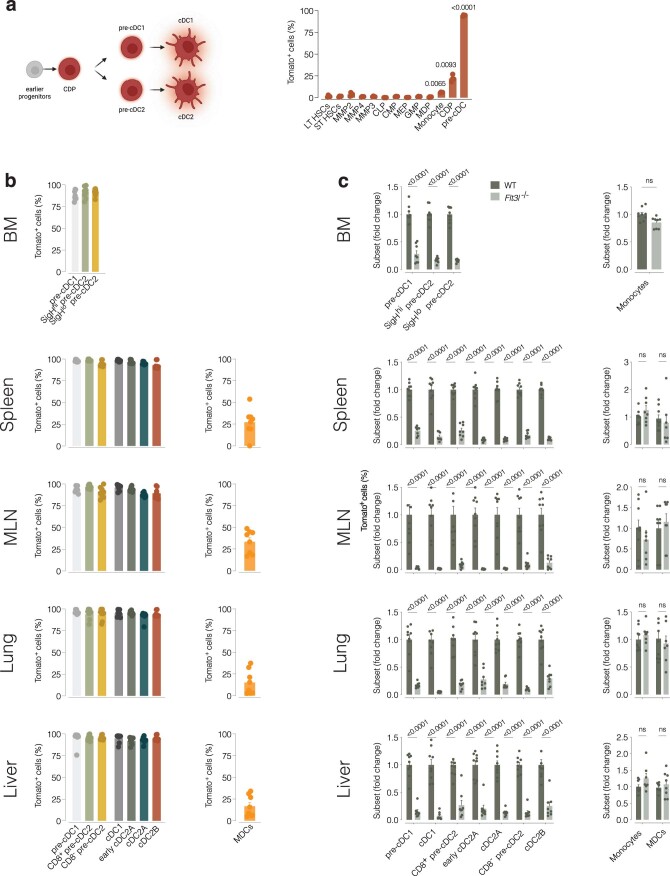
cDC2As and cDC2Bs are bona fide cDC subsets. **a**, (left) schematic depicting strategy for labelling of cDC lineages in DNGR-1 lineage tracer mice (*C9a*^*tdTOM*^). Figure was generated with BioRender. (right) FACS analysis showing % Tomato^+^ bone marrow progenitors identified as in reference^54^. **b**, FACS analysis showing % Tomato^+^ cells in the indicated cDC and pre-cDC subtypes and MDCs as reference for a poorly-labelled lineage. **c**, FACS analysis showing relative number of the indicated cDC and pre-cDC subtypes in WT versus Flt3L-deficient mice. Number of monocytes and MDCs from different tissues is also shown as reference for a Flt3L-independent lineage. Tissues analysed are indicated at the left of the graphs. Each dot represents one mouse (n = 8), and data were pooled from two experiments, in c data are expressed as fold-difference from WT (means ± SEM). Gating and quantifications come from UMAPs as shown in Extended Data Fig. 7b–d (see later) for the bone marrow and Extended Data Fig. 4c–e for the spleen, MLN, lung and liver. Monocytes and MDCs were identified as in ref. ^18^. Each dot represents one biological replicate (n = 8), and data are a pool of two experiments (means ± SEM). For panels (a, c) one-way ANOVA (with Tukey correction) was used for comparison of the groups against the labelling of MDPs or against the WT control. P values are indicated on top of the graphs.

Pre-cDC2s are too rare in other peripheral tissues to allow for sorting and adoptive transfer. In the spleen, MLN, lung and liver of T-bet-ZsGreen mice, we detected Esam^+^ cDC2As that expressed*Tbx21* transcripts (Extended Data Fig. 5d) and higher levels of T-bet-ZsGreen than CLEC12A^+^ cDC2Bs (Fig. 3d). The T-bet-ZsGreen signal in Ly6C^+^ pre-cDC2s was much lower than in cDC2As (Fig. 3d); however, it was detectable and significantly higher in CD8α^+^ pre-cDC2As than in CD8α^−^ pre-cDC2Bs across all tissues (Fig. 3d). Transfer of sorted spleen T-bet-ZsGreen^+^ pre-cDC2s and T-bet-ZsGreen^−^ pre-cDC2s into congenic mice indicated that T-bet-ZsGreen expression was retained (and increased) throughout the lifespan of cDC2As but not cDC2Bs and their progenitors^15^ (Extended Data Fig. 5e). At steady state, the ratio of T-bet-ZsGreen^+^ cDC2As to T-bet-ZsGreen^−^ cDC2Bs was greater in lymphoid tissues (Fig. 3e). Similarly, lymphoid tissues contained a larger proportion of pre-cDC2As, whereas pre-cDC2Bs predominated in nonlymphoid tissues (Fig. 3e). Finally, all these populations, in contrast to CD11b^hi^Ly6C^+^ monocytes or CD64^hi^CD88^+^ monocyte-derived cells (MDCs), displayed near-complete labeling in *Clec9a*^*Cre*^ lineage-tracing mice (*C9a*^*tdTomato*^) and were markedly reduced in frequency (85 ± 11%) in *Flt3l*^−/−^ mice (Extended Data Fig. 6a–c). This suggested the existence of two cDC2 lineages across tissues, both bona fide members of the cDC family.

### Two bone marrow pre-cDC2 subsets are related to cDC2As and cDC2Bs

**Fig. 4:**
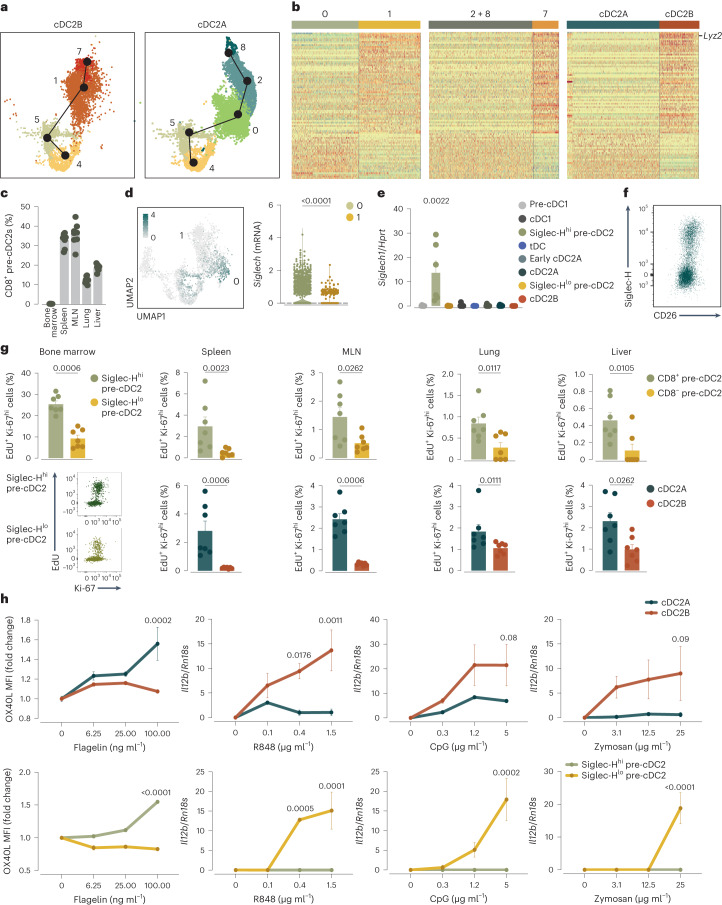
The bone marrow contains two populations of pre-cDC2s that can be segregated according to Siglec-H expression and are related to cDC2As and cDC2Bs. **a**, Pseudotime analysis of scRNA-seq data (Fig. 2b–d) from cluster 4 to clusters 7 and 8 concatenated from the bone marrow, spleen and lung. **b**, Heatmap of 87 DEGs between early pre-cDC2s (clusters 0 and 1) in the bone marrow (left), late pre-cDC2 clusters (clusters 2 and 8, and cluster 7) from the bone marrow, spleen and lung (middle) and comparison of our pre-cDC scRNA-seq data to those of splenic cDC2As and cDC2Bs from Brown et al.^15^ (right). Expression levels ranged from low (blue) to high (orange). **c**, Expression of CD8α on pre-cDC2s from the bone marrow, spleen, MLN, lung and liver, gated as in Extended Data Fig. 4c–e. **d**, *Siglech* expression projected on the scRNA-seq UMAP of bone marrow pre-cDCs as in Fig. 2a (left) and expression of *Siglech* in cluster 0 or 1 from bone marrow pre-cDCs (right). **e**, RT–qPCR for *Siglech* normalized to *Hprt* in spleen cDCs sorted as in Extended Data Fig. 1b and bone marrow pre-cDCs sorted as in Extended Data Fig. 7a. **f**, Representative flow cytometry plot showing Siglec-H and CD26 on pre-cDC2s from the bone marrow gated as single live Lin^−^CD11c^+^MHC-II^−/lo^CD11b^−/lo^SIRPα^−^CD135^+^CD43^+^Ly6C^+^ cells as in Extended Data Fig. 4b–d. **g**, 5-Ethynyl-2′-deoxyuridine (EdU) incorporation and Ki-67 staining on CD8α^−^ or CD8α^+^ (or Siglec-H^−^ or Siglec-H^+^ in the bone marrow) pre-cDC2s identified from the UMAP gates as in Extended Data Figs. 4e and 7d (top) and cDC2As and cDC2Bs identified from the UMAP gates as in Extended Data Fig. 4e from bone marrow, spleen, MLN, lung and liver. **h**, OX40L MFI and *Il12b* mRNA normalized to *Hprt* (RT–qPCR) in splenic cDC2As and cDC2Bs sorted as in Extended Data Fig. 1b and bone marrow Siglec-H^−^ or Siglec-H^+^ pre-cDC2s sorted as in Extended Data Fig. 8a after overnight culture with flagellin, R848, CpG or zymosan. In **c**,**e**,**g**, each dot represents one mouse (*n* = 3 in **h**, *n* = 6 in **e**, *n* = 7 in **g**, *n* = 8 in **c**). Data are from one of two experiments (**h**) or a pool of two (**c**,**e**,**g**) (mean ± s.e.m.; median ± IQR for the violin plot). A two-tailed Mann–Whitney *U*-test (**d**,**g**) or two-way analysis of variance (ANOVA) (with Tukey correction, **e**,**h**) was used to compare groups (in **e**, the comparison is relative to Siglec-H^lo^ pre-cDC2s). *P* values are indicated above the graphs.

Next, we investigated whether the lineage bias of pre-cDC2As and pre-cDC2Bs occurred as they entered the tissue or, as for pre-cDC1s and pre-cDC2s, before leaving the bone marrow. Pseudotime analysis of scRNA-seq data from bone marrow pre-cDCs suggested two mutually exclusive cDC2A and cDC2B differentiation trajectories (Fig. 4a). We compared the gene expression profiles of the cell clusters that defined the two trajectories (Fig. 4b). Among the transcripts that segregated clusters 0 and 1 in the bone marrow, we found 87 that overlapped with some of the transcripts that segregated late pre-cDC2As (clusters 2 and 8) and late pre-cDC2Bs (cluster 7) in the periphery, as well as those that segregated cDC2As and cDC2Bs (Fig. 4b and Supplementary Table 4). This overlap was statistically significant (*P* = 3.9 × 10^−42^; Fig. 4b), suggesting that specification toward cDC2As and cDC2Bs was already patent at the level of bone marrow pre-cDC2s.

**Extended Data Fig. 7:**
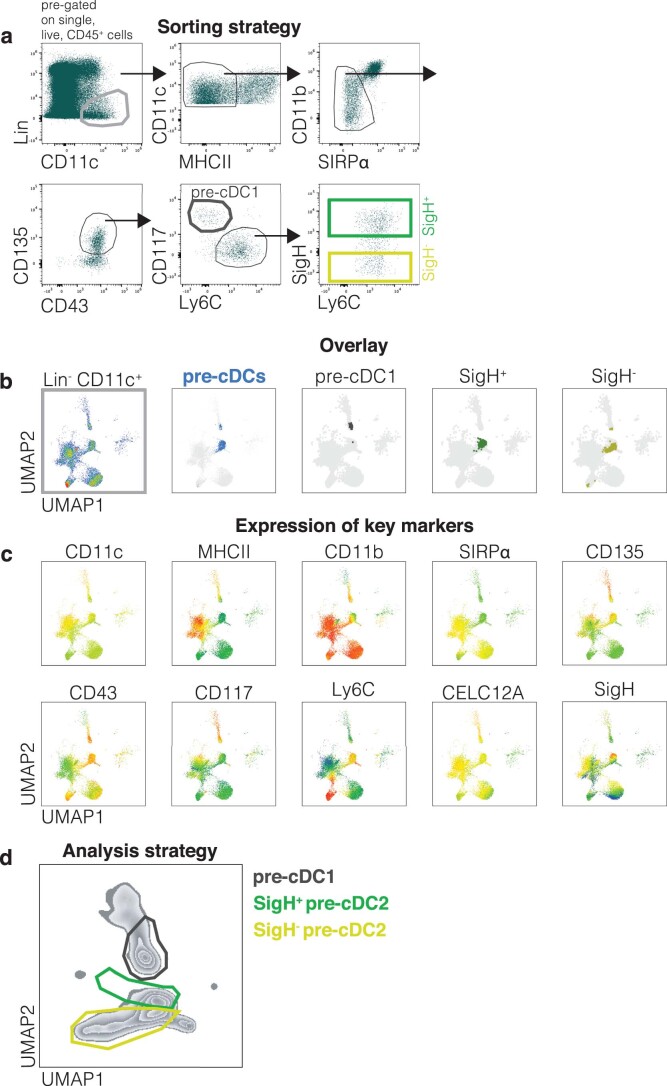
Pre-cDC subset identification in the bone marrow. **a**, Sorting strategy for bone marrow pre-cDC subsets. Leftmost panel has been pre-gated on single, live, CD45^+^, and lineage^−^ spleen cells. The lineage cocktail includes antibodies against CD3, Ly6G, SiglecF, B220, CD19, NK1.1, Ly6D, and Ter119. CD117 and Ly6C are used to identify pre-cDC1s (dark grey) and pre-cDC2s, respectively. SiglecH labels the putative pre-cDC2As (light green) whereas the putative pre-cDC2Bs are SiglecH^−^ (yellow). Arrows denote gate hierarchy. **b**, Manual gates used in a overlaid onto a UMAP analysis. The UMAP was generated using the Lin^−^ CD11c^+^ cells from the first gate of the manual strategy in a and used the following markers: CD11c, MHC-II, CD26, CD64, CD88, XCR1, SIRPα, ESAM, CLEC12A, CD11b, CD43, CD135, CD117, Ly6C, and SiglecH. **c**, The expression of key markers used to define different pre-cDC subpopulations in the UMAPs is shown in the form of heatmaps. Expression levels are represented as a colour gradient from low (blue) to high (orange). **d**, Analysis strategy for pre-cDC subsets in the bone marrow. The plot has been zoomed in the population of pre-cDCs shown in the second panel of b (highlighted in blue). Dark grey gate are pre-cDC1s, green gate are SiglecH^+^ pre-cDC2s and yellow gate are SiglecH^−^ pre-cDC2s.

**Extended Data Fig. 8:**
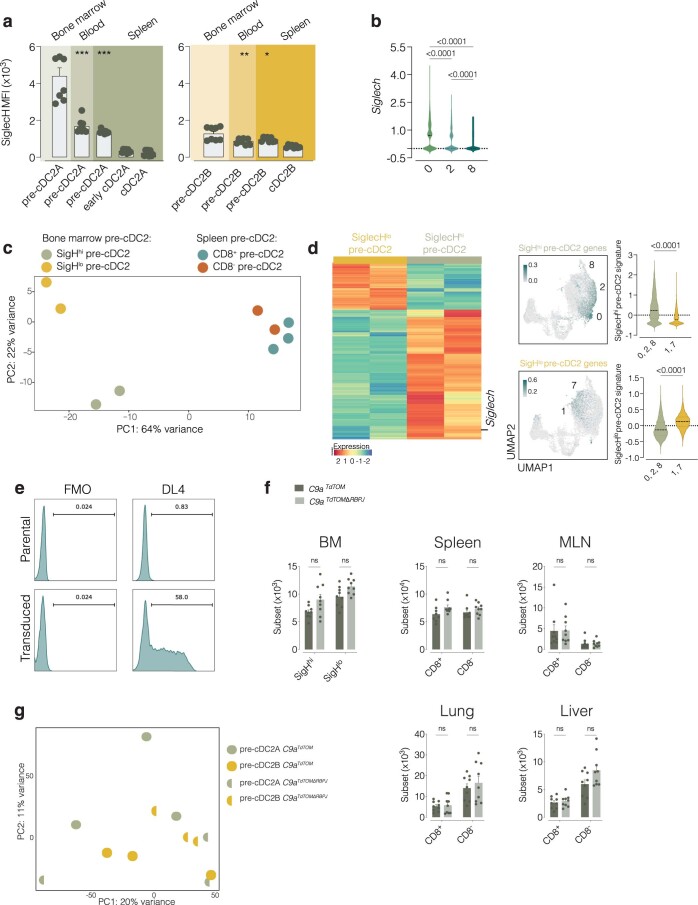
cDC2A differentiation trajectory post bone marrow egress. **a**, FACS analysis of SiglecH expression by the indicated pre-cDC2 or cDC2 populations isolated from the tissues indicated on top of the graphs. Gating is shown in Extended Data Fig. 7b–d for the bone marrow and Extended Data Fig. 4c–e for peripheral organs. **b**, Violin plot depicting the expression of *Siglech* in the clusters from the concatenated UMAP of the scRNAseq analysis (see Fig. 2a). **c**, PCA of all expressed genes from a bulk RNAseq of the indicated pre-DC2 populations from spleen (Sorted as shown in Extended Data Fig. 4a) and bone marrow (sorted as shown in Extended Data Fig. 7a). **d**, (left) Heatmap representation of the top DEGs (an adjusted p value of < 0.05) defining SiglecH^−^ pre-cDC2 and SiglecH^+^ pre-cDC2 analysed by bulk RNAseq (same analysis as c). Expression levels are represented as a colour gradient from low (blue) to high (orange). Each column represents a sample coming from a pool of 8 mice. (right) Feature plots representing the score of the DEGs (shown in the heatmap, used as signatures) of SiglecH^−^ and SiglecH^+^ pre-cDC2 on the concatenated UMAP. Expression levels are shown as a gradient from low (light grey) to high (teal). On the right is a violin plot depicting the expression of the DEG-derived signatures by the indicated clusters. **e**, FACS analysis of transduced OP9 cells showing overexpression of DL4. Sorted DL4^hi^ cells (bottom right panel) were used as feeder cells for Fig. 5a, b. **f**, FACS analysis showing the number of cells in the indicated pre-cDC2 populations from *C9a*^*tdTOM*^ (dark grey) or *C9a*^*TdTOMΔRBPJ*^ (light grey) mice. Gating is shown in Extended Data Fig. 7b–d for the bone marrow and Extended Data Fig. 4c–e for peripheral organs. **g**, PCA of all expressed genes from a new bulk RNAseq of pre-DC2 populations (same as 5c) sorted (as shown in Extended Data Fig. 7a) from the bone marrow of *C9a*^*TdTOM*^ versus *C9a*^*TdTOMΔRBPJ*^ mice. Each dot represents a sample coming from a pool of 3 mice. In panel a and f, each dot represents one mouse (n = 7 in a 9 in f), and data were a pool from two experiments (means ± SEM, median ± IQR for violin plot). Two-way ANOVA (with Tukey correction, a,b and f) or Mann-Whitney test (two-tailed, d) was used to compare the different groups. P values are indicated on top of the graphs.

In contrast to peripheral tissues, we did not detect expression of CD8α in any pre-cDC2s in the bone marrow (Fig. 4c). However, scRNA-seq and quantitative PCR with reverse transcription (RT–qPCR) analysis identified Siglec-H as a potential marker for the putative bone marrow pre-cDC2As in cluster 0 (Fig. 4d,e). Flow cytometry analysis confirmed that bone marrow pre-cDC2s could be segregated into Siglec-H^+^ and Siglec-H^−^ populations^30^ (Fig. 4f and Extended Data Fig. 7a–d). Siglec-H expression was very low in pre-cDC2s or cDC2s from peripheral tissues, such as the spleen (Extended Data Fig. 8a), suggesting that Siglec-H expression was lost as early pre-cDCs differentiated into late pre-cDCs that leave the bone marrow, which is consistent with previous reports^30^. Accordingly, scRNA-seq data analysis showed that *Siglech* expression was higher in cells in cluster 0 and lower in more differentiated pre-cDC2As in clusters 2 and 8 (Extended Data Fig. 8b). We sorted Siglec-H^+^ and Siglec-H^−^ pre-cDC2s from the bone marrow and performed bulk RNA-seq analysis to obtain a DEG signature for both populations (Extended Data Fig. 8c–d and Supplementary Table 5). When mapped onto the scRNA-seq UMAP, the signature of the Siglec-H^+^ pre-cDC2s highlighted cells in clusters 0, 2 and 8, whereas the signature of the Siglec-H^−^ pre-cDC2s highlighted cells in clusters 1 and 7 (Extended Data Fig. 8d). We further used principal component analysis (PCA) to probe the relationship between bone marrow Siglec-H^+^ pre-cDC2s and Siglec-H^−^ pre-cDC2s and the CD8α^+^ pre-cDC2As and CD8α^−^ pre-cDC2Bs found in the spleen. Principal component 1 segregated cells according to tissue, while principal component 2 split the cells according to subset (Extended Data Fig. 8c), indicating similarity between Siglec-H^+^ and CD8α^+^ pre-cDC2s and Siglec-H^−^ and CD8α^−^ pre-cDC2s.

Siglec-H^+^ pre-cDC2s displayed a greater proliferation index than Siglec-H^−^ pre-cDC2s, which was similar to the difference between cDC2As and cDC2Bs (Fig. 4g). cDC2As and Siglec-H^+^ pre-cDC2s responded more strongly to flagellin stimulation, whereas cDC2Bs and Siglec-H^−^ pre-cDC2s were more responsive to R848, CpG and zymosan (Fig. 4h). Bone marrow Siglec-H^+^ pre-cDC2As and Siglec-H^−^ pre-cDC2Bs displayed comparable labeling to bone marrow pre-cDC1s in *Clec9a*^*Cre*^ lineage-tracing mice (Extended Data Fig. 6a–b) and were Flt3L-dependent (Extended Data Fig. 6c), suggesting that they all descended from CDPs and not monocytes. These data showed that Siglec-H^+^ pre-cDC2s and Siglec-H^−^ pre-cDC2s in the bone marrow resemble peripheral cDC2As and cDC2Bs, respectively in terms of gene expression, proliferation capacity and pattern of responsiveness to innate immune stimuli^4,14,15^.

### Lymphotoxin and Notch ligands sustain pre-cDC2A specification

**Fig. 5:**
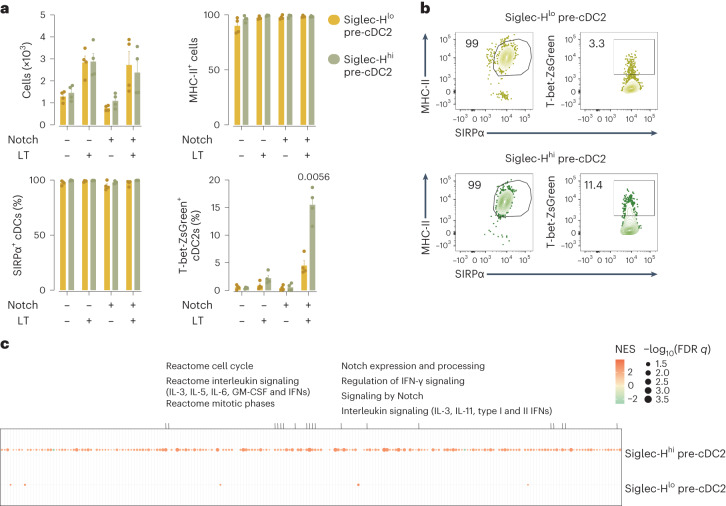
Bone marrow Siglec-H^+^ and Siglec-H^−^ pre-cDC2 populations respond differentially to lymphotoxin and Notch ligands to become cDC2s. **a**, Cell number, expression of MHC-II, expression of SIRPα and expression of T-bet-ZsGreen on bone marrow Siglec-H^lo^ pre-cDC2s and Siglec-H^hi^ pre-cDC2s after the culture of Siglec-H^+^ and Siglec-H^−^ pre-cDC2s sorted from the bone marrow of T-bet-ZsGreen mice (as in Extended Data Fig. 7a) with OP9 or OP9-DL4 stromal cells for 3 days in the presence of Flt3L with or without recombinant mouse lymphotoxin. **b**, Representative flow cytometry plots showing the expression of MHC-II, SIRPα and T-bet-ZsGreen on Siglec-H^+^ pre-cDC2s and Siglec-H^−^ pre-cDC2s on day 3 of coculture with OP9-DL4 stromal cells, Flt3L and lymphotoxin. **c**, GSEA analysis of bulk RNA-seq data in Siglec-H^hi^ pre-cDC2s and Siglec-H^lo^ pre-cDC2s sorted as in Extended Data Fig. 7a from *C9a*^*tdTomato*^ and *C9a*^*tdTomatoΔRBPJ*^ mice. Each dot represents one biological replicate (*n* = 4); data are a pool of two experiments (mean ± s.e.m.). FDR, false discovery rate; GM-CSF, granulocyte-macrophage colony-stimulating factor; IFN, interferon; NES, normalized enrichment score. In **a**, cells were analyzed using manual gating (as in **b**) and defined as: single; live; CD45.2^+^; CD11c^+^; and MHC-II^+^. cDC1s were defined as XCR1^+^, whereas cDC2s expressed SIRPα. A two-way ANOVA (with Tukey correction) was used for comparison. *P* values are indicated above the graphs.

We next sorted Siglec-H^+^ and Siglec-H^−^ pre-cDC2s from the bone marrow of T-bet-ZsGreen mice for in vitro differentiation assays. Both Siglec-H^+^ and Siglec-H^−^ pre-cDC2s cultured with Flt3L alone differentiated into cDC2s, as measured by the upregulation of MHC-II and SIRPα (Fig. 5a). However, they did not give rise to T-bet-ZsGreen^+^ cells unless cocultured with OP9-DL4 feeder cells, which provide Notch ligands (Extended Data Fig. 8e), in the presence of recombinant mouse lymphotoxin (Fig. 5a,b). In this setting, Siglec-H^+^ pre-cDC2s, but not Siglec-H^−^ pre-cDC2s, generated T-bet-ZsGreen^+^ cDC2As (Fig. 5a,b). This reiterated the importance of Notch signaling in the cDC2A differentiation pathway and led us to assess its effect on pre-cDC2s. Although *C9a*^*tdTomato*^ *and C9a*^*tdTomatoΔRBPJ*^ mice had equivalent numbers of Siglec-H^+^ and Siglec-H^−^ pre-cDC2s in the bone marrow and CD8α^+^ and CD8α^−^ pre-cDC2s in the periphery (Extended Data Fig. 8f), bulk RNA-seq analysis showed that bone marrow pre-cDC2s from *C9a*^*tdTomatoΔRBPJ*^ mice displayed an altered gene expression profile (Extended Data Fig. 8g and Supplementary Table 6). This was particularly noticeable for Siglec-H^+^ pre-cDC2s (Supplementary Table 6). Gene set enrichment analysis (GSEA) identified ‘signaling by Notch’, as well as cell cycle and cytokine receptor signaling as pathways altered in *C9a*^*tdTomatoΔRBPJ*^ Siglec-H^+^ pre-cDC2s (Fig. 5c). Thus, Notch signals were especially critical for the continued development of bone marrow Siglec-H^+^ pre-cDC2s.

### Pre-cDC2 subset specification starts in the bone marrow

**Fig. 6:**
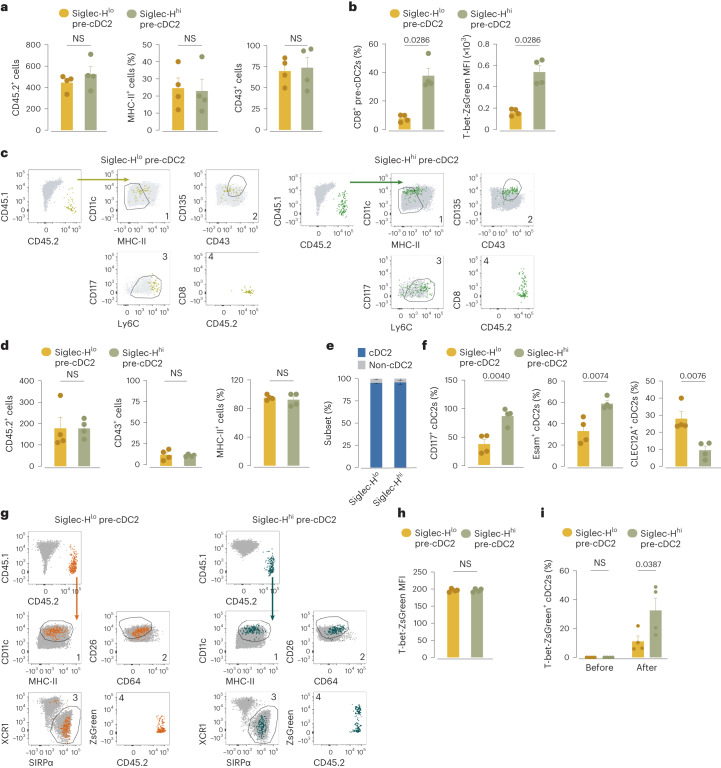
Pre-cDC2 specification toward the cDC2A versus cDC2B fate starts in the bone marrow. **a**, Number of cells and expression of MHC-II and CD43 on CD45.2^+^ cells recovered from the spleen of CD45.1 WT recipient mice 3 days after transfer of Siglec-H^−^ or Siglec-H^+^ pre-cDC2s isolated from T-bet-ZsGreen mice (sorted as in Extended Data Fig. 7a). **b**, Expression of CD8α (%) and T-bet-ZsGreen (MFI) on CD45.2^+^ cells isolated from T-bet-ZsGreen mice and recovered from CD45.1 WT mice as in **a**. **c**, Manual gating to confirm the UMAP analysis used for quantification in **a**,**b**. **d**, Number of cells and expression of MHC-II and CD43 on CD45.2^+^ cells recovered from the spleen of CD45.1 WT recipient mice 6 days after transfer of Siglec-H^−^ or Siglec-H^+^ pre-cDC2s isolated from T-bet-ZsGreen mice (sorted as in Extended Data Fig. 7a). **e**, cDC2 specification (as measured using SIRPα upregulation) of CD45.2^+^ cells isolated from T-bet-ZsGreen mice and recovered from CD45.1 WT mice as in **d**. **f**, Expression of CD117, Esam and CLEC12A (%) on CD45.2^+^ cells isolated from T-bet-ZsGreen mice and recovered from CD45.1 WT mice as in **d**. **g**, Manual gating to confirm the UMAP analysis used for quantification in **d**–**f**. **h**, T-bet-ZsGreen MFI on Siglec-H^−^ and Siglec-H^+^ pre-cDC2s from the bone marrow of T-bet-ZsGreen mice before transfer. Background autofluorescence was subtracted by gating on equivalent cells from WT mice. **i**, T-bet-ZsGreen^+^ (%) in Siglec-H^−^ or Siglec-H^+^ pre-cDC2s (or their progeny after transfer) isolated from the bone marrow of CD45.2 T-bet-ZsGreen mice before transfer or 6 days after transfer into CD45.1 WT mice (as in **d**). Each dot represents one mouse (*n* = 4, including **e**); data are a pool of two experiments (mean ± s.e.m.). A two-tailed Mann–Whitney *U*-test (**a**–**h**) or two-way ANOVA (with Tukey correction, **i**) was used to compare the fate of Siglec-H^−^ and Siglec-H^+^ pre-cDC2s. *P* values are indicated above the graphs. NS, not significant.

Next, we adoptively transferred Siglec-H^+^ or Siglec-H^−^ bone marrow pre-cDC2s from CD45.2 T-bet-ZsGreen mice into sublethally irradiated CD45.1 recipients. On day 3 after transfer, we recovered equivalent numbers of CD45.2^+^ cells from the spleens of both recipient groups and most were MHC-II^−/lo^CD43^+^ pre-cDCs (Fig. 6a). Siglec-H^+^ pre-cDC2s preferentially acquired CD8α and T-bet-ZsGreen expression, whereas Siglec-H^−^ pre-cDC2s remained negative for both markers (Fig. 6b,c). On day 6 after transfer, a time point that allowed for complete conversion of the transferred cells into cDC2s, virtually 100% of CD45.2^+^ cells were SIRPα^+^MHC-II^hi^CD43^−^ (Fig. 6d,e). Siglec-H^+^ pre-cDC2s preferentially gave rise to Esam^+^ or CD117^+^ cDC2s, whereas Siglec-H^−^ pre-cDC2s preferentially gave rise to CLEC12A^+^ cDC2s (Fig. 6f), confirming previous observations^30^. Even though neither bone marrow Siglec-H^+^ pre-cDC2s nor Siglec-H^−^ pre-cDC2s expressed detectable T-bet-ZsGreen at the time of the transfer, Siglec-H^+^ pre-cDC2s showed an increased tendency to give rise to T-bet-ZsGreen^+^ cDC2s (Fig. 6g–i). These experiments indicated that cDC2A and cDC2B lineage bias was already imprinted at the level of the pre-cDC2s that leave the bone marrow.

### Lineage tracing suggests distinct cDC2A and cDC2B ontogeny

**Fig. 7:**
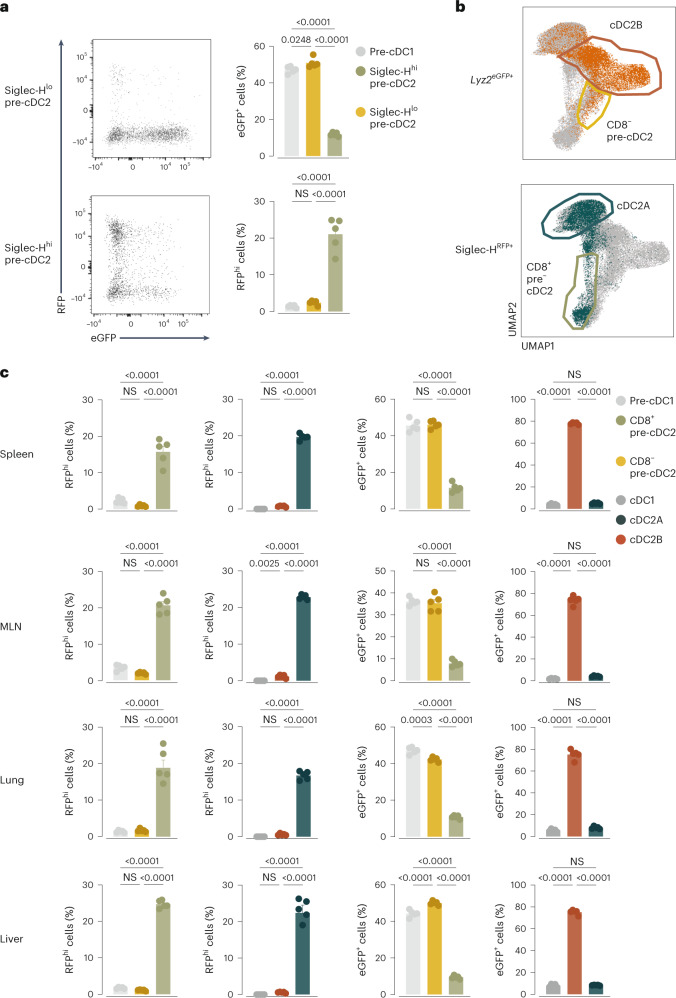
Lineage tracing confirms distinct cDC2A and cDC2B ontogenetic lineages. **a**, Representative flow cytometry plots of the expression of RFP and eGFP on Siglec-H^+^ and Siglec-H^−^ pre-cDC2s and percentage of Siglec-H-RFP^+^ and LysM-eGFP^+^ cells among pre-cDC1s and Siglec-H^−^ or Siglec-H^+^ pre-cDC2s from the bone marrow of *SigH*^*RFP*^*Lyz2*^*eGFP*^ mice. Pre-cDCs were identified using the UMAPs as in Extended Data Fig. 7b–d. **b**, Representative UMAPs (concatenated spleen, MLN, lung and liver) generated on CD11c^+^Lin^−^ cells using CD11c, MHC-II, CD26, CD64, CD88, XCR1, SIRPα, Esam, CLEC12A, CD11b, CD43, CD135, CD117, Ly6C and CD8α as in Extended Data Fig. 4c–e, overlaying RFP^hi^ and eGFP^hi^ cells in cDC2As and cDC2Bs and CD8α^−^ or CD8α^+^ pre-cDC2s. **c**, Percentage of RFP^+^ or eGFP^+^ cDC1s, cDC2As, cDC2Bs and pre-cDC1s, and CD8α^−^ pre-cDC2s or CD8α^+^ pre-cDC2s identified using the UMAPs as shown in Extended Data Fig. 4c–e from the spleen, MLN, lung and liver of *SigH*^*RFP*^*Lyz2*^*eGFP*^ mice. Gates for RFP^+^ and GFP^+^ cells were set using WT mouse cell counterparts. Each dot represents one mouse (*n* = 5); data are from one of two experiments (mean ± s.e.m.). A one-way ANOVA (with Tukey correction) was used for comparison. *P* values are indicated above the graphs.

**Extended Data Fig. 9:**
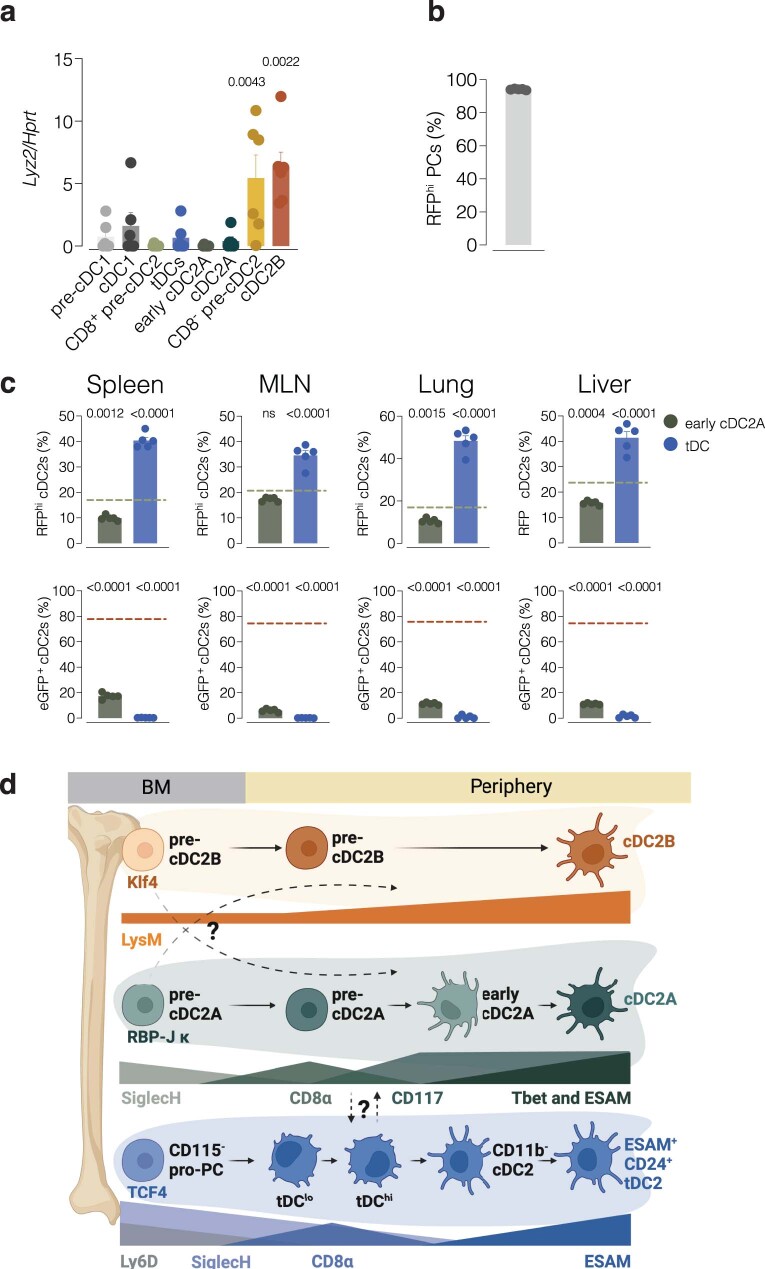
Model for cDC2A and cDC2B ontogeny. **a**, qRT-PCR analysis showing the expression of *Lyz2* in cDC, tDC and pre-cDC populations from the spleen (sorted as shown in Extended Data Figs. 1b and 4a). Data are normalised to housekeeping gene *Hprt*. **b**, FACS analysis showing the percentage of RFP^+^ in splenic plasmacytoid cells (defined as CD45.2^+^, Lin^+^, CD11c^+^, MHC-II^+^, SiglecH^+^, CD26^+^ CD64^−^ cells) from SiglecH lineage tracing (*SigH*^*RFP*^) mice crossed to *Lyz2*^*eGFP*^ reporter mice. **c**, FACS analysis showing the percentage of RFP^+^ (top) or eGFP^+^ (bottom) among early cDC2As or tDCs across the indicated organs. Gating is shown in 1b and Extended Data Fig. 1c–d. Dotted line is the reference value for RFP^+^ pre-cDC2A (top) or eGFP^+^ cDC2B (bottom) percentage in each tissue. Each dot represents one mouse (n = 5 in b and c and 6 in a), and data from one of two experiments (b-c) or pooled from two experiments (a) (means ± SEM). One-way ANOVA (with Tukey correction) was used to compare: in a, CD8α^−^ pre-cDC2 were compared against CD8α^+^ pre-cDC2 and cDC2B against cDC2A and in c, the tDCs and the early cDC2As (separately) with the pre-cDC2As (top) or the cDC2Bs (bottom). P values are indicated on top of the graphs. **d**, Schematic representation of a model for cDC2A and cDC2B ontogeny: In cDC2A differentiation, SiglecH-positive pre-cDC2As downregulate SiglecH as they leave the bone marrow and acquire the expression of CD8α as they colonise the tissues. Subsequent differentiation of these pre-cDC2As into tissue cDC2As involves downregulation of CD8α and upregulation of CD117 and MHC-II. T-bet expression is progressively upregulated throughout the entire cDC2A differentiation trajectory. cDC2A development is RBP-Jκ-dependent. In cDC2B differentiation, the bone marrow generates pre-cDC2Bs that express LYSM but lack SiglecH and CD8α. This population differentiates into cDC2Bs marked by increased LYSM tracing and upregulation of MHC-II and CLEC12A. cDC2B development is KLF4-dependent. The question marks denote the gaps that remail to be addresses in our model: Clonal analysis, as well as the use of better or additional markers will be necessary to assess the level of plasticity within bone marrow cDC2 progenitors (top question mark). Similarly, the split between the cDC2A and the tDC lineage remains to be confirmed by a genetic approach (bottom question mark). Figure was generated with BioRender.

To confirm these findings without cell transfer or irradiation, we used *Siglech*^*iCre*^*Rosa26*^*LSL-RFP*^ mice (hereafter *SigH*^*RFP*^), which trace the progeny of Siglec-H-expressing precursors^22^. In parallel, we sought to define pre-cDC2Bs and cDC2Bs independently of lack of expression of Siglec-H, CD8α or T-bet. Gene expression analysis of cDC2A versus cDC2B lineages (Fig. 4b and Extended Data Fig. 9a) suggested that LysM (*Lyz2*) might act as a marker for the latter. As such, we crossed the *SigH*^*RFP*^ mice to a *Lyz2*^*eGFP*^ reporter strain^42^ to generate *SigH*^*RFP*^*Lyz2*^*eGFP*^ mice. Plasmacytoid cells, which express Siglec-H^22^, were Siglec-H-red fluorescent protein (RFP)^+^ in these mice (Extended Data Fig. 9b). A high percentage (41 ± 7%) of tDCs were also Siglec-H-RFP^+^ (Extended Data Fig. 9c), which is consistent with the notion that they can express Siglec-H and descend from Siglec-H^+^ plasmacytoid cell precursors^25^. In the cDC lineage, Siglec-H-RFP labeling was found in bone marrow Siglec-H^+^ pre-cDC2s (21 ± 5%) but not Siglec-H^−^ pre-cDC2s (2.4 ± 0.6%) or pre-cDC1s (1.4 ± 0.3%) (Fig. 7a), while LysM-enhanced green fluorescent protein (eGFP) expression was found in Siglec-H^+^ pre-cDC2s (12 ± 1%), Siglec-H^−^ pre-cDC2s (51 ± 3%) and pre-cDC1s (47 ± 2%) (Fig. 7a). Even though Siglec-H expression was extinguished as pre-cDC2As left the bone marrow, the dichotomy was preserved across peripheral lymphoid and nonlymphoid organs: the frequency of Siglec-H-RFP^+^ cells was higher among tissue CD8α^+^ pre-cDC2s than in CD8α^−^ pre-cDC2Bs or pre-cDC1s (CD8α^+^ pre-cDC2s: 20 ± 4%; CD8α^−^ pre-cDC2s: 1.5 ± 0.5%; pre-cDC1s: 2.3 ± 1%), while the opposite was true for LysM-eGFP cells (CD8α^+^ pre-cDC2s: 10 ± 2%; CD8α^−^ pre-cDC2s: 43 ± 6%; pre-cDC1s: 43 ± 5%) (Fig. 7b,c). In the differentiated cDC2 compartment, Siglec-H-RFP labeling was largely restricted to Esam^+^ cDC2As and early cDC2As, mirroring the labeling of CD8α^+^ pre-cDC2As (Fig. 7b,c and Extended Data Fig. 9c). In contrast, LysM-eGFP expression was preferentially seen in CLEC12A^+^ cDC2Bs and was absent in cDC1s (Fig. 7b,c). These data were consistent with the notion that cDC2As and cDC2Bs were derived from distinct Siglec-H^+^ and LysM^+^ precursors (Extended Data Fig. 9d).

### Bone marrow specification of cDC2s is conserved across species

**Fig. 8:**
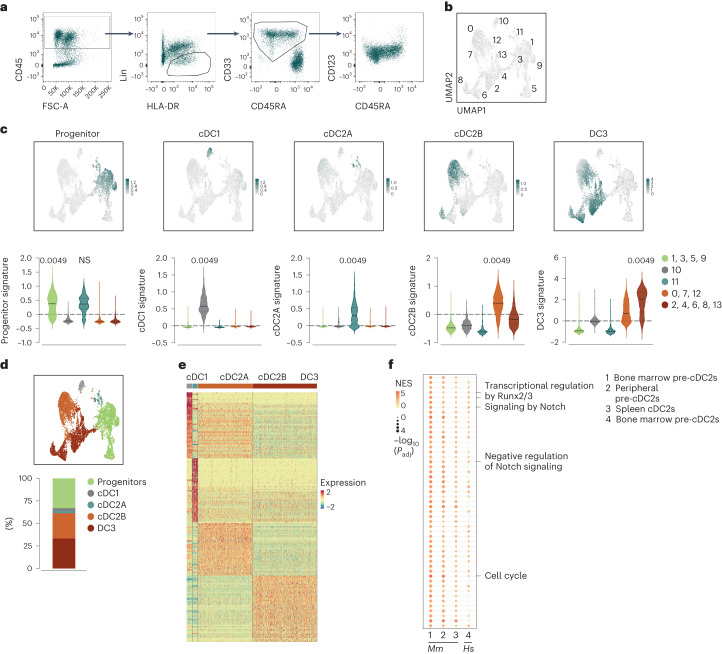
The bone marrow specification of the cDC2A and cDC2B lineages is conserved across species. **a**, Sorting strategy for human bone marrow cells isolated as CD45^+^CD3^−^CD14^−^CD15^−^CD16^−^CD19^−^CD20^−^HLA-DR^+^CD33^+^CD45RA^−^ cells (*n* = 3 human donors). The arrows denote the gate hierarchy. FSC-A, forward scatter area. **b**, UMAP displaying scRNA-seq analysis of cells in the CD33^+^ sorting gate depicted in **a** (*n* = 3 human donors). **c**, Feature plots representing the score for the gene expression signatures in CD34^+^ progenitors, cDC1s, cDC2As, cDC2Bs and DC3s projected onto the UMAP and violin plots of the scores within the cluster groups 1, 3, 5 and 9; 10; 11; 0, 7 and 12; and 2, 4, 6, 8, 13. Expression levels are shown as a gradient from low (light gray) to high (teal). **d**, Quantification of the proportion of CD34^+^ early progenitors and direct progenitors of cDC1s, cDC2As, cDC2Bs and DC3s found in the annotated UMAP (top). **e**, Heatmap representation of the top DEGs (*P*_adj _ < 0.05) defining the cDC1s, cDC2As, cDC2Bs and DC3s found in the annotated UMAP. Expression levels are represented as a color gradient from low (blue) to high (orange). **f**, GSEA analysis showing significantly modified pathways in mouse bone marrow pre-cDC2As versus pre-cDC2Bs as in Fig. 2 (1), mouse peripheral pre-cDC2As versus pre-cDC2Bs as in Fig. 2 (2) and mouse splenic cDC2As versus cDC2Bs from Brown et al.^15^ (3), with human cDC2A versus cDC2B lineages from the bone marrow (4). In **c**, a one-way ANOVA (with Tukey correction) was used for comparison (median ± IQR). Comparisons are from one group of clusters relative to all other groups and indicated when not significant. Reference groups are (from left to right): 1, 3, 5, 9; 10; 11; 0, 7, 12; and 2, 4, 6, 8, 13. *P* values are indicated above the graphs.

**Extended Data Fig. 10:**
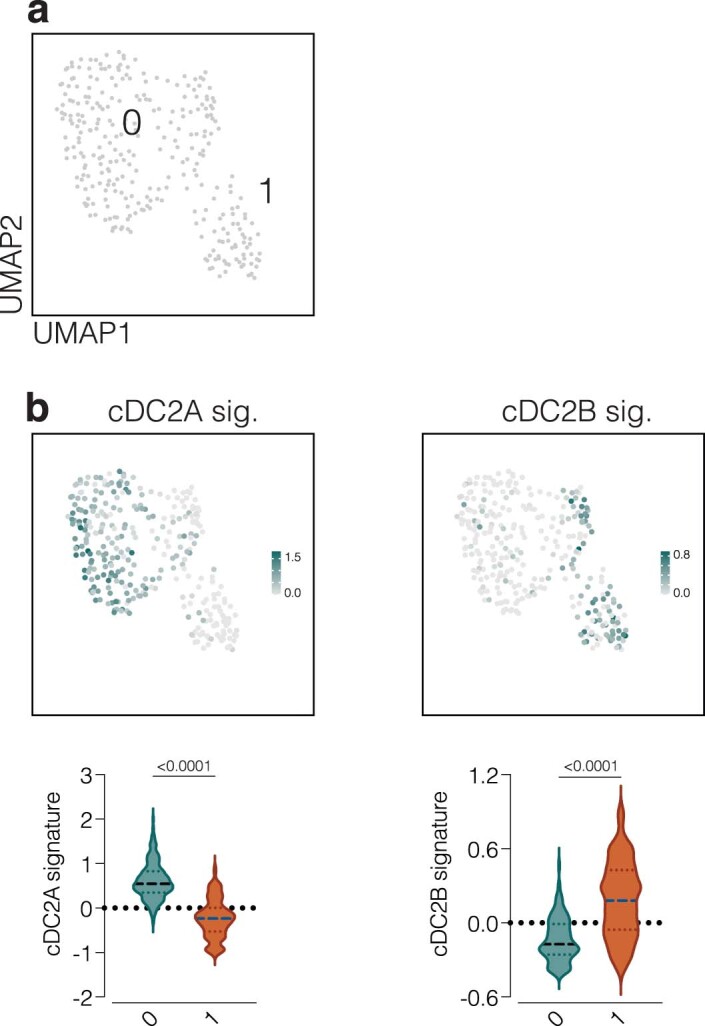
Identification of pre-cDC2A and pre-cDC2B in human spleen. **a**, UMAP of data taken from Ref. ^15^ displaying a single cell analysis of human splenic pre-cDCs with unsupervised clustering. **b**, Feature plots representing the score for gene expression signatures of cDC2A and cDC2B (Extended Data Fig. 9, from^15^) projected onto the UMAP space. Expression levels are shown as a gradient from low (light grey) to high (teal). Below are violin plots depicting the expression of the two gene signatures by the indicated clusters in the x axes. Mann-Whitney test (two-tailed) was used for comparisons (median ± IQR). P values are indicated on top of the graphs.

We reanalyzed a published dataset that reported cDC2As and cDC2Bs among HLA-DR isotype (HLA-DR)^+^ cells from human spleen^15^. We identified a small cluster of HLA-DR^+^ pre-cDCs that could be further segregated into two clusters resembling cDC2As or cDC2Bs (Extended Data Fig. 10a,b), suggesting that human spleen contained pre-cDC2As and pre-cDC2Bs. To assess if these pre-cDC2s can also be found in bone marrow, we purified them using a gating strategy previously developed for human blood cDCs and their precursors^21^. CD3^−^CD14^−^CD15^−^CD16^−^CD19^−^CD20^−^CD45^+^HLA-DR^+^CD45RA^−^CD33^+^ cells sorted from the bone marrow of human donors (Fig. 8a) were subjected to scRNA-seq analysis. After excluding a small number of contaminants, we generated a UMAP that included 8,240 cells and 14 clusters (Fig. 8b). We used the signatures of all previously identified DC populations in humans, including cDC1, cDC2A, cDC2B and DC3 (refs. ^15,21,23^) (Supplementary Table 7) to annotate the clusters and included a progenitor signature^43^ (Supplementary Table 7) to visualize the differentiation directionality. Earlier progenitors were found in clusters 1, 3, 5 and 9 while cluster 10 contained the pre-cDC1/cDC1 lineage (Fig. 8c). Cluster 11 showed the highest score for the cDC2A signature whereas pre-cDC2Bs/cDC2Bs were found in clusters 0, 7 and 12 and DC3s in clusters 2, 4, 6, 8 and 13 (Fig. 8c). Overall, we found three distinct populations of pre-cDCs/cDCs (cDC1, cDC2A and cDC2B), and DC3s^19,39^ (Fig. 8d,e and Supplementary Table 8). Notably, GSEA comparing mouse cDC2 lineages alongside human pre-cDC2A/cDC2A (cluster 11) and pre-cDC2B/cDC2B (clusters 0, 7 and 12) showed a considerable overlap in pathways that were enriched in the cDC2A lineage across species (Fig. 8f). Thus, the cDC2A/cDC2B subset specification appears conserved across mice and humans.

## Discussion

Distinct cell types or different cell states can contribute to the heterogeneity of cDC2s. In this study, we identified pre-cDC2s in mouse bone marrow and peripheral tissues that displayed differential propensity to generate cDC2As versus cDC2Bs and could account for previously described cDC2 types. Much like the separation between cDC1s and cDC2s, the specification of cDC2As and cDC2Bs started in the bone marrow. These data argue for a model in which cDC subsets (cDC1, cDC2A and cDC2B) and related lineages (DC3s, plasmacytoid cells, tDCs) are prespecified in the bone marrow and constitute bona fide DC subsets rather than tissue-determined cell states.

We could not ascertain whether pre-cDC2As and pre-cDC2Bs are unipotential as we noted residual capacity of bone marrow Siglec-H^+^ or spleen CD8α^+^ pre-cDC2 to generate cDC2Bs. This might reflect plasticity but could equally represent technical limitations in cell sorting or in the penetrance of Cre-mediated recombination in lineage tracing. In addition, some of the output cells in our lineage-tracing experiments, and in vivo transfer and in vitro differentiation assays, did not express markers that allowed us to assign them to either the cDC2A or cDC2B lineages. Clonal analysis, as well as more extensive phenotyping, will be important in the future to distinguish precursor bias from absolute commitment. Siglec-H^+^ and Siglec-H^−^ pre-cDC2s are proposed to represent distinct developmental stages of cDC2s^30^. We further found a population of bone marrow pre-cDC2s that never expressed Siglec-H and generates cDC2Bs. We also showed that Siglec-H^+^ pre-cDC2As lost the expression of Siglec-H as they left the bone marrow, concomitant with the acquisition of CD8α expression and before final differentiation into cDC2As in tissues. This is consistent with a previous report that Siglec-H^+^ pre-cDC2s can give rise to cDC2s^15,17,30^ but argues that it is the case only for cDC2As and not cDC2Bs.

Specific organ niches can drive adult monocytes to become resident macrophages akin to those that colonized the organs during embryonic life^44^. In this setting, tissue signals override ontogeny to specify myeloid cell fate. However, unlike tissue macrophages that can live up to 18 months in mice and 11 years in humans^45^, the lifespan of cDCs in mouse tissues is estimated to be 3–6 days in most organs^27,46^. This might explain why cDC2 subsets are prespecified in the bone marrow, as they may not have enough time to be ‘instructed’ by their niche. However, this does not negate the importance of the tissue microenvironment^15,31,34,47^ as we showed that pre-cDC2s required a permissive setting to complete their differentiation. Different environmental cues in lymphoid versus nonlymphoid organs could modulate the proliferation and lifespan of pre-cDC2 types or their progeny, explaining the contrasting proportion of cDC2As and cDC2Bs in these organs. In line with this notion, Esam^+^ cDC2As proliferate more than Esam^−^ cDC2s in response to lymphotoxin expressed by splenic ILC3s^4,48,49^. Differential expression of chemokine receptors in pre-cDC2As versus pre-cDC2Bs (for example, Ccr1, Ccr2 and Ccr9, as noted in our scRNA-seq analysis) could additionally affect the tropism of pre-cDC subsets toward different organs.

We focused on ontogeny and gene expression as the primary tool for cDC definition, as done by others^17,25^. It has been suggested that progenitors that express Siglec-H^+^ and share other markers with plasmacytoid cells (most likely corresponding to the pre-cDC2As described in this study) act as cDC2 precursors^17^. tDCs can generate Esam^+^ cells that show phenotypic overlap with, yet are distinct from, cDC2As^25^. Our data suggest that pre-cDC2As display phenotypic similarities to tDCs, but arise from Ly6D^−^ precursors, display distinct gene expression signatures from tDCs, can be distinguished by higher expression of SIRPα, MHC-II, CLEC12A and CD43 and lower expression of CD24, and display lower labeling than tDCs in *SigH*^*RFP*^ mice. As such, our data are consistent with the notion that tDCs and pre-cDC2As represent distinct populations, although we note that both can give rise to Esam^+^ DCs (this work and Sulczewski et al.^25^). Based on the expression of CD11b and CD24, tDC-derived Esam^+^ DCs may not be canonical cDC2As, although expression of T-bet remains to be assessed. Finer delineation of the cDC2A and the tDC lineages will require a genetic approach, such as hCD2 or CD300c lineage-tracing mice.

DC3s have recently been shown to be distinct from cDCs and monocytes and arise from Ly6C^+^ monocyte-DC progenitors that do not go through a pre-cDC stage^39^. Similarly, tDCs originate from Ly6D^+^ bone marrow progenitors shared with plasmacytoid DCs^25^. The discovery of ontogenetically distinct DC3s, tDCs, together with our observations, supports a model in which the bone marrow is the original site of DC precursor bias toward the cDC1, cDC2A, cDC2B, DC3 and tDC fate. Additional studies will be necessary to establish the degree of plasticity in pre-cDC commitment during inflammation and assess the functional properties of progeny cDC2As, cDC2Bs, DC3s and tDCs.

## Methods

### Ethics

The research in this manuscript complies with all relevant ethical regulations. Mouse experiments were planned in accordance with the principles of the three Rs (replacement, reduction, refinement). All experiments were performed in accordance with the United Kingdom Animals (Scientific Procedures) Act of 1986. The UK Home Office accredited all researchers for animal handling and experimentation. Dispensation to carry out animal research at the Francis Crick Institute was approved by the institutional ethical review body and granted by the UK Home Office under PPL PF40C0C67.

### Mice

C57BL/6J (CD45.1^+^), C57BL/6J (CD45.2^+^), T-bet-ZsGreen^35^ (Taconic Biosciences), *Rbpj*^*loxP*/*loxP*^^50^ (abbreviated to *ΔRBPJ*), *Clec9a*^*Cre28*^ (abbreviated to *C9a*), *Flt3l*^−/−^ (Taconic Biosciences), *Rosa26*^*LSL-tdTomato*^ (The Jackson Laboratory) mice were bred at the Francis Crick Institute in specific pathogen-free conditions. *Siglech*^*iCre*^ mice^22^ (B6-*Siglech*^*tm1(iCre)Ciphe*^) were generated by the Centre d’Immunophénomique (Marseille, France) and crossed to the *Rosa26*^*LSL-RFP*^^51^ and *Lyz2*^*eGFP*^^42^ strains. All genetically modified mouse lines were backcrossed to C57BL/6J; 6–12-week-old male and female mice were age-matched and sex-matched in all experiments.

### Human bone marrow

Human bone marrow was purchased from STEM CELL Technologies and processed as described previously^52^. Briefly, cells from three independent donors (female aged 31 years, and males aged 29 and 24 years) were thawed in prewarmed FCS containing DNase I (10 μg ml^−1^), washed and stained for fluorescence-activated cell sorting (FACS) as described below (the antibodies used for staining are listed in Supplementary Table 10). After sorting, human pre-DCs and DCs from the three individuals were pooled to minimize individual variability before submission for scRNA-seq.

### Preparation of single-cell suspensions

Mice were perfused intracardially through the left ventricle using cold PBS before tissue collection. Livers were further perfused in situ via the portal vein. This procedure efficiently removed circulating cells as assessed by injection of CD45 antibody (intravenously) 2 min before tissue collection and processing^40^. Spleens, MLNs, lungs and livers were cut into small pieces and digested with collagenase VIII (1 mg ml^−1^, Sigma-Aldrich) and DNase I (0.4 mg ml^−1^, Roche) in Roswell Park Memorial Institute (RPMI) 1640 medium for 15 min (spleen and MLN) or 25 min (lung and liver) at 37 °C. Digested tissues were passed through a 70-μm cell strainer (BD Biosciences) and washed with FACS buffer (3% FCS and 5 mM EDTA in PBS). For lung and liver, leukocytes were enriched using Percoll gradient centrifugation (GE Healthcare) as described previously^18^. For bone marrow, the femur, tibia and hip extremities were cut and spun for 30 s at 10,000 r.p.m. Cells were resuspended in FACS buffer after centrifugation. For the transfer assays, the spine and humerus were also collected and crushed with a mortar before collecting a cell suspension with a micropipette and filtering using a 100-μm cell strainer.

### Pre-cDC enrichment and isolation

Single-cell suspensions from the bone marrow, spleen and lung were enriched for pre-cDCs by staining for lineage-restricted markers with biotin-conjugated or fluorescein isothiocyanate (FITC)-conjugated antibodies (CD3, Ly6G, Siglec-F, B220, CD19, Ly6D, NK1.1 and Ter119) and depleting T, B and plasmacytoid cells, as well as red blood cells, neutrophils, eosinophils and their precursors, using the EasySep Mouse Biotin Positive Selection Kit II (STEMCELL Technologies). Cells were stained as described below. Pre-cDC and cDC subsets were FACS-sorted on an Aria Fusion (BD Biosciences) with a 100-μm nozzle using the gating strategy shown in Extended Data Figs. 1b, 3a, 4a and 7a as indicated.

### Flow cytometry analysis

Cells were preincubated with blocking anti-CD16/32 in FACS buffer for 10 min at 4 °C and then stained for 40 min at 4 °C with an antibody cocktail and LIVE/DEAD Fixable Dead Cell Stain Kit (Thermo Fisher Scientific) in FACS buffer. Lineage (Lin) markers included CD3, Ly6G, Siglec-F, B220, CD19, Ly6D, NK1.1 and Ter119, unless otherwise specified. The antibodies used for flow cytometry are listed in Supplementary Table 10. Samples were acquired using a BD FACSymphony A5 (BD Biosciences) or in an ID7000 (Sony Biotechnology) or SpectroFlo Aurora (Cytek) spectral analyzers. Data were analyzed using FlowJo (v.10.8.2) as shown in Extended Data Figs. 1, 4 and 8. UMAP analysis^53^ of the flow cytometry data was carried out on the basis of CD11b, CD11c, CD26, CD43, CD64, CD88, CD135, SIRPα, MHC-II, CD117, Ly6C, Siglec-H, CD8α, XCR1, CLEC12A and Esam expression. Annotation of clusters on the UMAP plots was done by using defining markers for each immune population. Validation of the accuracy of the UMAP analysis versus manual gating was confirmed by overlaying different immune populations identified by either strategy. Monocytes and MDCs were identified as in Cabeza-Cabrerizo et al.^18^. Earlier bone marrow progenitors were identified as in Cardoso et al.^54^.

### scRNA-seq

Mouse and human pre-cDCs (viability >95%) were processed according to the manufacturer’s instructions on a 10X Genomics Chromium platform. Library generation was performed using the Chromium Single Cell 3′ Reagents Kits (10X Genomics) and sequenced on an HiSeq 4000 (Illumina) to achieve an average of approximately 63,000 reads per cell and approximately 4,000 cells per sample. Raw reads were initially processed using the Cell Ranger v.3.0.2 pipeline^55^, which deconvolved reads to their cell of origin using the unique molecular identifier tags, aligned these to the mm10 transcriptome (to which we added the eGFP sequence (https://www.addgene.org/browse/sequence/305137/) to detect GFP-expressing cells) using STAR (v.2.5.1b)^56^ and reported cell-specific gene expression count estimates. All subsequent analyses were performed in R v.3.6.1 using the Seurat (v.3) package^57^. Genes were considered to be ‘expressed’ if the estimated (log_10_) count was at least 0.1. Primary filtering was then performed by removing from consideration cells expressing fewer than 50 genes and cells for which mitochondrial genes made up greater than three standard deviations from the mean of mitochondria-expressed genes. PCA decomposition was performed and, after consideration of the eigenvalue ‘elbow-plots’, the first 30 components were used to construct the UMAP plots per sample. Multiple samples were integrated using 2,000 variable genes and Seurat’s canonical correlation analysis. Cluster-specific gene markers were identified using a Wilcoxon rank-sum test; the top 10 or 20 genes ranked according to log fold change per cluster were used to generate a heatmap. Clusters were annotated using known marker genes and gene signatures (refs. ^15,30^ and Supplementary Table 9). Contamination with plasmacytoid cells and MDCs was ruled out by integrating our data with previous scRNA-seq analysis that included these cells^22,58^ and checking for cluster segregation. GSEA was used to identify pathways enriched in a cluster or a group of them against others. CytoTRACE^59^ was used to determine the differentiation states of cells. Trajectories were identified using the package Slingshot (v.1.4.0)^60^, using the undifferentiated cluster as a starting point and the dimensionality reduction UMAP coordinates. Lineages were identified showing different trajectories ending in specific differentiated cells (Supplementary Table 11). Comet analysis^41^ was used to identify putative flow cytometry markers for populations defined using scRNA-seq. The analysis was performed by loading the scRNA-seq data, the UMAP and the clustering from Seurat on the Comet portal^41^).

### Bulk RNA-seq

Pre-cDCs and cDCs (gating strategy shown in Extended Data Figs. 1b, 4a and 7a) were FACS-sorted from the bone marrow and spleen either from WT or *C9a*^*tdTomato*^ and *C9a*^*tdTomatoΔRBPJ*^ mice. Cells (0.6 × 10^4^ to 3.2 × 10^4^) were sorted directly into lysis buffer to avoid loss of material. RNA was extracted using the RNeasy Mini Kit (QIAGEN). The NuGEN Ovation RNA-Seq System (V2) was used for complementary DNA (cDNA) synthesis followed by the NuGEN Ultralow Library System (V2) for library preparation. Samples were normalized to 1 ng of RNA for input; the preparation was performed according to the manufacturer’s guidelines. Sequencing was performed on an Illumina HiSeq 4000, with 100-base pair single-end reads. After sequencing, samples were normalized and analyzed. GSEA was used to identify pathways enriched in cells from different genotypes.

### RNA extraction and RT–qPCR

Cells were collected in RLT buffer and RNA extraction was performed using the RNeasy Micro Kit (QIAGEN). cDNA synthesis was carried out using SuperScript II Reverse Transcriptase (Invitrogen). RT–qPCR was performed using the TaqMan Universal PCR Master Mix (Thermo Fisher Scientific) and primers (Supplementary Table 12). Analysis was performed on a QuantStudio PCR system (Thermo Fisher Scientific) using Δ^Ct^ quantification.

### Pre-cDC differentiation assays and OP9 transduction

OP9 cells were obtained from ATCC (CRL-2749). The OP9 DL1/GFP^61^ line was obtained from the Francis Crick Institute Cell Services. To generate a feeder cell line overexpressing DL4, we made use of a commercial lentiviral system (Lenti ORF clone of Dll4 (Myc-DDK-tagged), OriGene). Vesicular stomatitis virus G (VSV G)-pseudotyped lentivirus was generated by transfecting HEK 293T cells with 1.3 μg of pCMV delta R8.2 (Addgene), 0.6 μg of VSV G (Addgene) and 1.3 μg of transfer plasmid (OriGene). Supernatant was collected 72 h after transfection, spun down to remove debris and used to transduce OP9 cells (CRL-2749, ATCC). After 24 h, cells were selected with puromycin and subsequently FACS-sorted to enrich for DL4-expressing cells (Extended Data Fig. 8e).

Flt3L-driven differentiation of pre-cDCs was carried out as outlined elsewhere^18^. Briefly, pre-cDCs were cocultured with OP9 cells^62^ into 96-well plates in RPMI 1640 medium supplemented with l-glutamine (Gibco), penicillin-streptomycin (Gibco), nonessential amino acids (Gibco), HEPES (Gibco), sodium pyruvate (Gibco), 10% FCS (Sigma-Aldrich) and β-mercaptoethanol (Gibco) (R10). Then, 2 × 10^4^ OP9, OP9-DL1/GFP^61^ or OP9-DL4 cells were plated; the following day, 1 × 10^3^ to 5 × 10^3^ sorted pre-cDCs from T-bet-ZsGreen mice were added to the OP9 monolayer after removing the medium and replacing it with fresh medium containing mouse Flt3L (300 ng ml^−1^) or lymphotoxin (10 ng ml^−1^) (R&D Systems). Progeny cells were assessed 3 days later using flow cytometry. DC differentiation was assessed according to MHC-II upregulation, whereas plasmacytoid cell differentiation was quantified according to the expression of B220 and Siglec-H. cDC1s were defined as XCR1^+^, and cDC2s were defined as SIRPα^+^. cDC2A fate was assessed using T-bet upregulation (ZsGreen expression).

### Pre-cDC2 and cDC2 stimulations

Pre-cDC2 (Siglec-H high and low) and cDC2 populations were sorted from the bone marrow and spleen, respectively (gating strategy shown in Extended Data Figs. 1b and 7a). Subsequently, 0.5–1 × 10^4^ cells were cultured in R10 in the presence and absence of different stimuli (InvivoGen) at varying concentrations: flagellin (6–100 ng ml^−1^), R848 (0.1–1.5 μg ml^−1^), CpG ODN 1668 (0.3–5 μg ml^−1^) and zymosan (3–25 μg ml^−1^). After 12 h of culture, cells were recovered for subsequent FACS analysis (OX40L) or processed for RT–qPCR (as outlined above). The viability of recovered cells was similar across cell types and treatments, as assessed using flow cytometry.

### Cell transfers

The cell transfer experiments were performed as described before^18^. Briefly, spleen and bone marrow (legs, hip bone, spine and humerus) were collected from CD45.2 C57BL/6J (WT or Tbx21-ZsGreen) mice. Pre-cDC2s subsets were sorted as indicated in Extended Data Figs. 4a and 7a. Cells (10,000–40,000) were injected intravenously into sublethally irradiated (6.6 Gy) CD45.1 C57BL/6J mice 1 day after irradiation. Spleen cells were analyzed 3 or 6 days after transfer.

### Proliferation assessment

Mice were injected intraperitoneally with 1 mg EdU (Lumiprobe) 2 h before tissue collection for assessment of cell proliferation. EdU detection was carried out using the Click-iT Plus EdU Alexa Fluor 647 Flow Cytometry Kit (Thermo Fisher Scientific) after surface staining and fixation and permeabilization. Intranuclear staining of Ki-67 was performed in parallel to EdU detection. Cells were analyzed using flow cytometry as described above.

### Statistical analysis and reproducibility

No statistical methods were used to predetermine sample sizes but our sample sizes were similar to those reported in previous publications^18^. Mice were not randomized in cages, but each cage was randomly assigned to a treatment group. Investigators were not blinded to mouse identity during necropsy and sample analysis. Male and female mice were used to perform the experiments. However, we did not observe differences between sexes. In all cases measurements were taken from distinct samples and no individual data points were excluded under any circumstances. Statistical analyses were performed using Prism 9 (GraphPad Software). Results are depicted as the mean ± s.e.m. and median ± IQR in violin plots. The statistical test used is specified in each figure legend. For pair comparisons, a nonparametric two-tailed Mann–Whitney *U*-test was used. When ANOVA was used, Tukey correction was performed. Data distribution was assumed to be normal, but this was not formally tested. For Tables 1, 3 and 5, a two-sided Wald test with Benjamini–Hochberg correction was used. For Supplementary Tables 2, 4, 6 (DEGs) and 8, a one-sided Wilcoxon signed-rank test with Benjamini–Hochberg correction was used. For Supplementary Table 6 (enrichment), a one-sided hypergeometric test with Benjamini–Hochberg correction was used. For Supplementary Table 11, a one-sided Wald test not corrected for multiple testing was used. These comparisons were made using the DESeq2. Genes with *P*_adj_ < 0.05 were taken forward and used to draw a heatmap using the ComplexHeatmap R package or to generate a gene signature.

### Reporting summary

Further information on research design is available in the Nature Portfolio Reporting Summary linked to this article.

## Online content

Any methods, additional references, Nature Portfolio reporting summaries, source data, extended data, supplementary information, acknowledgements, peer review information; details of author contributions and competing interests; and statements of data and code availability are available at 10.1038/s41590-024-01745-9.

### Supplementary information

Reporting Summary

Supplementary Table 1 DEGs obtained using bulk RNA-seq analysis among splenic cDC2As and cDC2Bs from mice.

Supplementary Table 2 Comet analysis listing putative surface markers for clusters 0, 1, 2, 3, 6 and 7 of the scRNA-seq analysis of pre-cDCs from the bone marrow, spleen and lung of mice.

Supplementary Table 3 DEGs obtained using bulk RNA-seq among splenic CD8α^−^ and CD8α^+^ pre-cDC2s from mice.

Supplementary Table 4 DEGs between early pre-cDC2 clusters (clusters 0 and 1) in the bone marrow, late pre-cDC2s clusters (clusters 2, 8 and 7) from the bone marrow, spleen and lung and comparison of the pre-cDC scRNA-seq data to those of splenic cDC2As and cDC2Bs from ref. 15.

Supplementary Table 5 DEGs obtained using bulk RNA-seq analysis among bone marrow Siglec-H^−^ and Siglec-H^+^ pre-cDC2s from mice.

Supplementary Table 6 Enrichment analysis (and DEGs) from bulk RNA-seq comparing bone marrow Siglec-H^−^ versus Siglec-H^+^ pre-cDC2s from *C9a*^*tdTomato*^ and *C9a*^*tdTomatoΔRBPJ*^ mice.

Supplementary Table 7 Gene signatures used to annotate the UMAP from the scRNA-seq of human DC progenitors isolated from the bone marrow of donors.

Supplementary Table 8 DEGs among groups of UMAP clusters (scRNA-seq of human bone marrow DC progenitors) encompassing immediate progenitors of cDC1, cDC2A, cDC2B and DC3.

Supplementary Table 9 Gene signatures used to annotate the UMAP from the scRNA-seq of pre-cDCs isolated from the bone marrow, spleen and lung of mice.

Supplementary Table 10 List of antibodies used in this study.

Supplementary Table 11 Change in genes in a pseudotime analysis (Slingshot) following the 4, 5, 1 and 7 (cDC2B lineage) or the 4, 5, 0, 2, 8 (cDC2A lineage) trajectories from our scRNA-seq of pre-cDCs isolated from the bone marrow, spleen and lung of mice.

Supplementary Table 12 List of TaqMan probes used in this study.

## Data Availability

The scRNA-seq and bulk RNA-seq data have been deposited in the Gene Expression Omnibus under accession nos. GSE217328, GSM6711828, GSM6711829, GSM6711830 and GSE244346. All other data needed to evaluate the conclusions in the manuscript are presented in the manuscript or the Supplementary Information.
