# Supplementary material for: Distinct ontogenetic lineages dictate cDC2 heterogeneity
Source: Nat Immunol. 2024 Feb 13;25(3):448–61. doi: 10.1038/s41590-024-01745-9 (PMC10907303; doi:10.1038/s41590-024-01745-9)
Supplement: Supplementary file 1 — Reporting Summary [file 41590_2024_1745_MOESM1_ESM.pdf]

Corresponding author(s): Caetano Reis e Sousa and Carlos M. Minutti

Last updated by author(s): 04/01/2024

Reporting Summary

Nature Portfolio wishes to improve the reproducibility of the work that we publish. This form provides structure for consistency and transparency in reporting. For further information on Nature Portfolio policies, see our Editorial Policies and the Editorial Policy Checklist.

Statistics

For all statistical analyses, confirm that the following items are present in the figure legend, table legend, main text, or Methods section.

n/a	Confirmed	
☐	☒	The exact sample size (*n*) for each experimental group/condition, given as a discrete number and unit of measurement
☐	☒	A statement on whether measurements were taken from distinct samples or whether the same sample was measured repeatedly
☐	☒	The statistical test(s) used AND whether they are one- or two-sided<br>*Only common tests should be described solely by name; describe more complex techniques in the Methods section.*
☒	☐	A description of all covariates tested
☐	☒	A description of any assumptions or corrections, such as tests of normality and adjustment for multiple comparisons
☐	☒	A full description of the statistical parameters including central tendency (e.g. means) or other basic estimates (e.g. regression coefficient) AND variation (e.g. standard deviation) or associated estimates of uncertainty (e.g. confidence intervals)
☐	☒	For null hypothesis testing, the test statistic (e.g. *F*, *t*, *r*) with confidence intervals, effect sizes, degrees of freedom and *P* value noted<br>*Give P values as exact values whenever suitable.*
☒	☐	For Bayesian analysis, information on the choice of priors and Markov chain Monte Carlo settings
☒	☐	For hierarchical and complex designs, identification of the appropriate level for tests and full reporting of outcomes
☐	☒	Estimates of effect sizes (e.g. Cohen's *d*, Pearson's *r*), indicating how they were calculated

Our web collection on statistics for biologists contains articles on many of the points above.

Software and code

Policy information about availability of computer code

Data collection	N/A
Data analysis	Single cell RNA sequencing analyses were performed in R v.3.6.1 (https://www.R-project.org/) using the Seurat (v3) package. Differentiation trajectories were identified using the package 'Slingshot' (version 1.4.0). Comet analysis was used to identify putative flow cytometry markers from populations defined by scRNAseq. Statistical analyses were performed using GraphPad Prism 9 software (GraphPad). Flow cytometry data was analysed using FlowJo (10.8.2). Samples were acquired using BD FACSDiva in a Symphony A5 (BD), or SA3800 in a ID7000 5L (SONY) or SpectroFlo in a 5L Aurora (Cytek) spectral analysers.

For manuscripts utilizing custom algorithms or software that are central to the research but not yet described in published literature, software must be made available to editors and reviewers. We strongly encourage code deposition in a community repository (e.g. GitHub). See the Nature Portfolio guidelines for submitting code & software for further information.

Data

Policy information about availability of data

All manuscripts must include a data availability statement. This statement should provide the following information, where applicable:

- Accession codes, unique identifiers, or web links for publicly available datasets
- A description of any restrictions on data availability
- For clinical datasets or third party data, please ensure that the statement adheres to our policy

Single cell and bulk RNA-seq data have been deposited in GEO under accession numbers GSE217328, GSM6711828, GSM6711829, GSM6711830. All other data needed to evaluate the conclusions in the paper are present in the paper or the Supplementary Materials.

Human research participants

Policy information about studies involving human research participants and Sex and Gender in Research.

Reporting on sex and gender	Human bone marrow was purchased from Stem Cell Technologies from male (2) and female (1) donors.
Population characteristics	Human marrows were selected from adults with similar age to minimise potential variability due to aging. The donors were 24 (male), 29 (male) and 31 (female) years old.
Recruitment	N/A
Ethics oversight	Human bone marrow was purchased from Stem Cell Technologies. The study was approved by the Francis Crick Institute Ethical Review Body.

Note that full information on the approval of the study protocol must also be provided in the manuscript.

Field-specific reporting

Please select the one below that is the best fit for your research. If you are not sure, read the appropriate sections before making your selection.

☒ Life sciences ☐ Behavioural & social sciences ☐ Ecological, evolutionary & environmental sciences

For a reference copy of the document with all sections, see nature.com/documents/nr-reporting-summary-flat.pdf

Life sciences study design

All studies must disclose on these points even when the disclosure is negative.

Sample size	No statistical methods were used to pre-determine sample sizes but our sample sizes are similar to those reported in previous publications (Cabeza-Cabrerizo, et al. 2021, Science Immunology.
Data exclusions	No individual data points were excluded under any circumstances.
Replication	All experiments were performed at least twice and only data that reproduced across all experiments was included in this manuscript.
Randomization	Mice were not randomized in cages, but each cage was randomly assigned to a treatment group.
Blinding	Investigators were not blinded to mouse identity during necropsy and sample analysis.

Reporting for specific materials, systems and methods

We require information from authors about some types of materials, experimental systems and methods used in many studies. Here, indicate whether each material, system or method listed is relevant to your study. If you are not sure if a list item applies to your research, read the appropriate section before selecting a response.

March 2021

Materials & experimental systems

n/a	Involved in the study
☐	☒ Antibodies
☐	☒ Eukaryotic cell lines
☒	☐ Palaeontology and archaeology
☐	☒ Animals and other organisms
☒	☐ Clinical data
☒	☐ Dual use research of concern

Methods

n/a	Involved in the study
☒	☐ ChIP-seq
☐	☒ Flow cytometry
☒	☐ MRI-based neuroimaging

Antibodies

Antibodies used	All antibodies, and their information (conjugate, clone, commercial house and working concentration) are listed in Supplementary figure 9
Validation	All antibodies used in this study are commercial and validated by selling companies. Working concentrations were determined by titration using the starting with the concentrations suggested by the commercial house.

Eukaryotic cell lines

Policy information about cell lines and Sex and Gender in Research

Cell line source(s)	OP9, OP9-DL1 and OP9-DL4 cells were acquired from the Cell Services facility of The Francis Crick Institute.
Authentication	All cell lines used in this study have been tested for Species ID only - this identified the lines as mouse cells
Mycoplasma contamination	All cell lines tested negative for mycoplasma contamination
Commonly misidentified lines (See ICLAC register)	N/A

Animals and other research organisms

Policy information about studies involving animals; ARRIVE guidelines recommended for reporting animal research, and Sex and Gender in Research

Laboratory animals	C57BL/6J (CD45.1+), C57BL/6J (CD45.2+), Tbx21-ZsGreen (Taconic Biosciences), Rbpj-fl (abbreviated ΔRBPJ), Clec9aCre (abbreviated C9a), Flt3l–/– (Taconic Biosciences), Rosa26-LSL-tdTomato (abbreviated tdTOM; the Jackson Laboratory) mice were bred at the Francis Crick Institute in specific pathogen–free conditions. SiglechiCre mice (B6-Siglechtm1(iCre)Ciphe) were generated by Centre d'Immunophénomique, Marseille, France and crossed to the Rosa26-LSL-RFP and the Lyz2-eGFP strains. All genetically modified mouse lines were backcrossed to C57BL/6J. Six- to 12-week-old male and female mice were age- and sex-matched in all experiments. The housing conditions are the following: Light cycles fluctuate from 7am-7pm. Temperature range from 20-24 degrees C and humidity is 55% +/- 10%.
Wild animals	No wild animals were used in the study.
Reporting on sex	Male and female mice were used to perform the experiments. However, we did not observe differences between sexes.
Field-collected samples	No field collected samples were used in the study.
Ethics oversight	All experiments were performed in accordance with the United Kingdom Animals (Scientific Procedures) Act of 1986. The UK Home Office accredited all researchers for animal handling and experimentation. Dispensation to carry out animal research at the Francis Crick Institute was approved by the Institutional Ethical Review Body and granted by the UK government Home Office; as such all research was carried under the project license PF40C0C67.

Note that full information on the approval of the study protocol must also be provided in the manuscript.

Flow Cytometry

Plots

Confirm that:

☒ The axis labels state the marker and fluorochrome used (e.g. CD4-FITC).

☒ The axis scales are clearly visible. Include numbers along axes only for bottom left plot of group (a 'group' is an analysis of identical markers).

☒ All plots are contour plots with outliers or pseudocolor plots.

☒ A numerical value for number of cells or percentage (with statistics) is provided.

Methodology

Sample preparation	Cells were preincubated with blocking anti-CD16/32 in FACS buffer for 10 min at 4°C and then stained for 40 min at 4°C with antibody cocktail and LIVE/DEAD Fixable Dead Cell Stain Kit in FACS buffer. Lineage (Lin) markers included CD3, Ly6G, SiglecF, B220, CD19, Ly6D, NK1.1, and Ter119. Antibodies used for flow cytometry are listed in Table S6.
Instrument	Samples were acquired using a Symphony A5 (BD), or a 5L ID7000 (SONY) or 5L Aurora (Cytek) spectral analysers.
Software	Data were analysed using FlowJo 10.
Cell population abundance	At least 5-10 x106 cells were stained and analysed so as to achieve a robust number of cells in the pre-cDC gate.
Gating strategy	UMAP analysis of flow cytometry data was generated on the basis of CD11b, CD11c, CD16/32, CD26, CD43, CD64, CD88, CD135, SIRPα, MHC-II, CD117, Ly6C, SiglecH, CD8α, XCR1, CLEC12A, and ESAM expression. Annotation of clusters on the UMAP plots was done by using defining markers for each immune population. The accuracy of our manual gating was confirmed on the UMAPs by overlaying different immune populations identified with manual gating.

☒ Tick this box to confirm that a figure exemplifying the gating strategy is provided in the Supplementary Information.
